# Preliminary study on comparative non-targeted metabolomics analysis sheds light on the chemical diversity of citrus fruit pulps

**DOI:** 10.1371/journal.pone.0353350

**Published:** 2026-07-22

**Authors:** Mingxia Wen, Bei Huang, Naveed Ahmad, Jiayan Wu

**Affiliations:** 1 Institute of Citrus Research, Zhejiang Academy of Agriculture Sciences, Taizhou, China; 2 Institute for Safflower Industry Research of Shihezi University/Pharmacy College of Shihezi University/Key Laboratory of Xinjiang Phytomedicine Resource and Utilization, Ministry of Education, Shihezi, China; 3 College of Mechanical Engineering, Donghua University, Shanghai, China; Universidad Autonoma de Chihuahua, MEXICO

## Abstract

Citrus flavor and nutritional quality are closely tied to metabolite composition, yet comparative metabolic dissection of fruit pulp traits across citrus subspecies remains insufficient. Here, we applied non-targeted LC–MS/MS metabolomics to examine chemical diversity in the fruit pulp of three representative citrus varieties: *Citrus reticulata* ‘Hongju 418’, *Citrus aurantium* ‘Changshan-huyou,’ and *Citrus junos* ‘Hunan Xiangcheng.’ Through differential metabolite analysis, multivariate modeling, correlation network construction, and KEGG pathway enrichment, we characterized the extent and nature of metabolic divergence among these genotypes. PCA, PLS-DA, and OPLS-DA revealed distinct metabolic clusters, underscoring strong genotype-specific variation. More than 300 differentially expressed metabolites were identified, including flavonoid glycosides, organic acids, phenolic derivatives, and limonoids. Hongju 418 was enriched in flavonoid biosynthetic pathways, Xiangcheng accumulated higher concentrations of organic and amino acids, and Huyou displayed a unique hybrid profile marked by elevated fatty acid and purine metabolism. Correlation and KEGG analyses consolidated these observations, revealing coordinated pathway-level shifts that define subspecies-specific metabolic architectures. Collectively, this work deepens current understanding of citrus pulp chemotypes and provides a robust biochemical foundation for advances in citrus breeding, quality assessment, and functional product innovation.

## 1. Introduction

Citrus fruits, members of the Rutaceae family, are among of the most economically and nutritionally important fruit groups worldwide [1,2]. Species such as *Citrus reticulata* (mandarins), *Citrus sinensis* (sweet orange) [3], and hybrids like *Citrus aurantium* (Changshan-huyou) are cultivated for their desirable sensory attributes and rich contents of vitamin C, flavonoids, antioxidants, and essential oils [4]. The pulp, which constitutes the primary edible portion, is widely consumed in fresh and processed forms and serves as a major dietary source of health-promoting metabolites [5]. Its chemical composition varies substantially across varieties and subspecies due to genetic and environmental factors, giving rise to differences in fruit quality, nutritional value, and bioactive potential [6–8]. Despite the commercial relevance of citrus pulp, comparative metabolomic studies at the varietal or subspecies level remain limited. Existing research has largely emphasized peels or juices, leaving the pulp—the most consumed fraction—relatively underexplored across diverse citrus genotypes [9–12].

Metabolomics provides a comprehensive approach to characterizing fruit quality by profiling both primary and secondary metabolites. In Citrus, key metabolite groups such as flavanones, phenolic acids, organic acids, and sugars contribute to sensory attributes, nutritional properties, and resilience to environmental stresses [13]. Secondary metabolites also function in defense against oxidative stress, UV radiation, and pathogens, and their synthesis is influenced by genotype, tissue specificity, and developmental stage [14,15]. Moreover, many citrus-derived metabolites have recognized pharmacological activities, including antioxidant, anti-inflammatory, and cardioprotective effects, underscoring their relevance to functional food and nutraceutical development [16,17]. Citrus flavonoids such as naringenin, eriocitrin, and diosmin exemplify compounds with strong radical-scavenging activity and notable regulatory effects on metabolic and inflammatory pathways [18,19]. Consequently, metabolomics plays an increasingly central role in citrus breeding, enabling the identification of traits associated with fruit quality, stress tolerance, and postharvest behavior [20,21], especially when combined with transcriptomic and genomic data to reveal genotype–phenotype associations and metabolic biomarkers [22].

Conventional targeted analyses using high-performance liquid chromatography (HPLC), gas chromatography–mass spectrometry (GC-MS), and nuclear magnetic resonance (NMR) have characterized major citrus flavonoids and volatiles [23–25], yet they often miss low-abundance or novel metabolites. Advances in non-targeted liquid chromatography–tandem mass spectrometry (LC-MS/MS) now allow broad and sensitive detection of diverse polar and semi-polar metabolites, offering deeper insights into citrus fruit chemistry [26,27]. Although recent applications have focused on citrus peels and juices, including responses to biotic and abiotic stresses [26,28], systematic metabolomic investigations of citrus pulp across subspecies remain scarce. Additionally, integrative analyses that combine multivariate statistics and pathway enrichment to identify subspecies-specific metabolic features are notably lacking.

Notably, this knowledge gap is particularly acute for commercially relevant varieties of citrus; although some genotypes underpin distinct functional food and nutraceutical industries, their pulp metabolomes remain largely uncharacterized at the subspecies level. ‘Hongju 418’ (*Citrus reticulata* ‘Hongju 418’), a leading loose-skin mandarin in China, is increasingly processed for flavonoid extraction and antioxidant dietary supplements [29,30]. ‘Huyou’ (*Citrus aurantium* ‘Changshan-huyou’) underpins a diverse industry spanning beverages, preserved fruits, and medicinal foods with documented anti-obesity and hepatoprotective bioactivities [31]. ‘Xiangcheng’ (*Citrus junos* ‘Hunan Xiangcheng’) is commercially transformed into functional powders and flavoring additives, with recent enzymatic modifications enhancing its dietary fiber content and anti-adipogenic potential [32,33]. Nevertheless, systematic comparative metabolomic investigations of their pulp tissues—the primary consumed fraction—remain fragmentary, impeding genotype-specific quality control and value-added functional food innovation.

This study addresses these knowledge gaps by performing a comprehensive non-targeted LC-MS/MS metabolomic analysis of fruits pulp tissues from three citrus varieties: ‘Hongju 418’, ‘Huyou’, and ‘Xiangcheng’. The aims are to (i) characterize the metabolite profiles of each variety, (ii) identify differentially accumulated metabolites among subspecies, and (iii) elucidate enriched biochemical pathways contributing to varietal diversity. Through multivariate statistical analysis, pathway enrichment, and metabolite correlation network construction, this study provides a metabolic framework that deepens understanding of citrus subspecies diversity and supports breeding, pulp quality assessment, and future multi-omics advancements.

## 2. Materials and methods

### 2.1. Plant material and sample preparation

**Fig 1:**
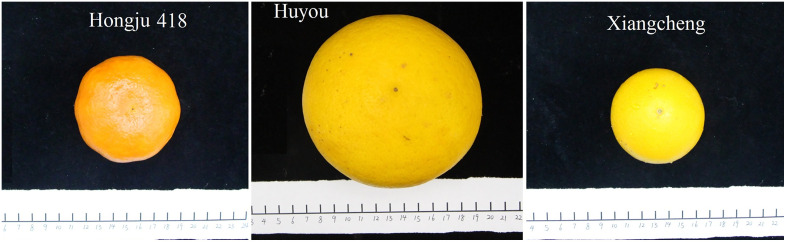
The photographs of the fruits used in this study.

All plant materials were cultivated under standard field conditions at the experimental station of the Zhejiang Citrus Research Institute, Taizhou, Zhejiang Province, China. The trees were grown using uniform agricultural practices, including routine irrigation, fertilization, pest management, and pruning, in accordance with local agronomic guidelines for citrus production. Fruits were harvested at the commercial maturity stage during the same growing season. On November 26th, 10 fruits were collected for each variety. At the same elevation line in the canopy, fruits from the east, south, west, north and center directions were respectively taken, with 2 fruits from each direction. The fruits sizes were basically the same. Immediately after collection, the pulps were carefully separated, then divided into 5 groups, constituting 5 repetitions. Flash-frozen in liquid nitrogen to prevent metabolic degradation, and stored at –80°C until metabolite extraction and LC-MS/MS analysis. All sampling was conducted in the morning hours (08:00–10:00 AM) to minimize variability due to diurnal fluctuations in metabolite levels. Photos of the fruits of three citrus varieties at their mature stages are shown in Fig 1. Although five biological replicates were initially prepared for each variety, stringent QC-based outlier screening resulted in three qualified replicates being used for the final multivariate statistical analysis (see Section 2.3.3 for exclusion criteria).

### 2.2. Metabolite extraction

Citrus pulp samples (approximately 100 mg each) were first ground into a fine powder under liquid nitrogen using a chilled mortar and pestle to preserve metabolite integrity. The powdered tissue was allowed to stand at 4°C for 4 hours, then centrifuged at 8,000 rpm to remove cellular debris. A 400 μL aliquot of each clarified sample was then mixed with 200 μL of pre-cooled ultrapure water and 800 μL of pre-chilled methanol/acetonitrile (1:1, v/v) to initiate metabolite extraction. The mixture was vortexed thoroughly and subjected to ultrasonic disruption in an ice bath for 60 minutes to enhance extraction efficiency. To precipitate proteins and other macromolecules, the extracts were incubated at –20°C for 1 hour, followed by centrifugation at 16,000 g for 30 minutes at 4°C. The resulting supernatant was carefully transferred and vacuum-dried in a high-speed centrifugal evaporator until complete solvent removal. For LC-MS/MS analysis, the dried residue was reconstituted in 100 μL of acetonitrile–water solution (1:1, v/v), vortexed, and centrifuged again at 16,000 g for 30 minutes at 4 °C to remove any remaining particulates. The final supernatant was transferred into LC-MS vials for instrumental analysis. Additionally, quality control samples were prepared by pooling equal volumes from each sample and analyzed periodically throughout the LC-MS/MS run to assess analytical stability and reproducibility.

### 2.3. LC-MS/MS analysis

#### 2.3.1. Chromatographic separation.

Metabolite separation was performed using an Agilent 1290 Infinity UHPLC system (Agilent Technologies, USA) equipped with a Waters ACQUITY UPLC BEH Amide column (1.7 µm, 2.1 × 100 mm), which is optimized for hydrophilic interaction liquid chromatography (HILIC). During the analysis, all sample vials were maintained at 4°C in the autosampler to ensure consistent temperature and sample integrity. The column temperature was set to 25°C, the flow rate was maintained at 0.3 mL min^-1^, and the injection volume for each sample was 5 μL. The chromatographic separation was carried out using a binary gradient elution system. Mobile phase A consisted of HPLC-grade water containing 25 mM ammonium acetate and 25 mM ammonia, while mobile phase B was acetonitrile. The gradient program was as follows: 0–0.5 minutes, 95% B; 0.5–7 minutes, B linearly decreased from 95% to 65%; 7–9 minutes, B decreased from 65% to 40%; 9–10 minutes, B held constant at 40%; 10–11.1 minutes, B increased linearly back to 95%; and 11.1–17 minutes, B was maintained at 95% for column re-equilibration. Quality control samples prepared by pooling equal aliquots from all experimental samples were injected at regular intervals to monitor the stability and reproducibility of the analytical system throughout the run.

#### 2.3.2. Mass spectrometry acquisition.

Mass spectrometric analysis was performed using a Triple TOF 5600 + mass spectrometer (AB SCIEX, USA) equipped with an electrospray ionization (ESI) source. Each sample was analyzed in both positive and negative ionization modes. The ESI source was operated under the following conditions: ion source gases 1 and 2 were both set to 60 psi, the curtain gas was set to 30 psi, and the source temperature was maintained at 600 °C. The ion spray voltage floating (ISVF) was set to ±5500 V for both positive and negative ion modes. The mass spectrometer was operated in data-dependent acquisition mode with information-dependent acquisition (IDA) in high-sensitivity mode. The full-scan mass range was set from m/z 60–1200 for TOF MS and from m/z 25–1200 for product ion scans. The accumulation time was 0.15 seconds per MS1 spectrum and 0.03 seconds per MS2 spectrum. The de-clustering potential (DP) was set to ±60 V, and the collision energy (CE) was fixed at 30 eV. The IDA acquisition excluded isotopes within 4 Da and allowed up to 6 candidate ions to be monitored per cycle.

#### 2.3.3. Data preprocessing and multivariate analysis.

Raw MS data files were converted into a compatible format and processed with MS-DIAL (version 4.90). The preprocessing workflow included peak detection, retention time alignment, peak area extraction, and feature annotation. Structural identification of metabolites was conducted based on accurate mass matching (tolerance < 25 ppm), isotopic pattern recognition, and MS/MS spectral matching against publicly available databases such as HMDB and MassBank, as well as an in-house reference library. Following feature extraction, ion peaks with more than 50% missing values across samples were removed to improve data robustness. Quality control-based outlier screening. Prior to multivariate statistical modeling, biological replicates were subjected to rigorous quality-control screening. Samples were excluded as outliers if they met any of the following criteria: (i) total ion chromatogram (TIC) base drift exceeding 20% or abnormal peak shape; (ii) missing values >50% across all detected features after retention time alignment; or (iii) falling outside the 95% confidence interval of Hotelling’s T^2^ in the unsupervised PCA score plot. As a result, three high-quality biological replicates per variety were retained for subsequent PCA, PLS-DA, and OPLS-DA analyses. Datasets from positive and negative ionization modes were then merged. The resulting data matrix was subjected to multivariate statistical analysis using SIMCA-P software (version 14.1, Umetrics, Umeå, Sweden). All variables were Pareto-scaled prior to statistical modeling. Principal component analysis (PCA) was used for unsupervised clustering to assess overall data structure, while supervised models, including partial least squares discriminant analysis (PLS-DA) and orthogonal PLS-DA (OPLS-DA), were used to identify differentially accumulated metabolites among the citrus varieties. Model performance and validity were evaluated using cross-validation and 200-permutation testing to avoid overfitting.

### 2.4. KEGG pathway enrichment analysis

Significantly different metabolites were mapped to biological pathways using the Kyoto Encyclopedia of Genes and Genomes (KEGG) database [5]. Enrichment analysis was performed using custom R scripts provided by Shanghai Bioprofile, with visualization through bar and bubble plots based on the rich factor, p-value, and metabolite count per pathway. Pathway significance was evaluated using adjusted p-values (FDR correction).

## 3. Results

### 3.1. Metabolite profiling across the selected citrus varieties

Non-targeted UPLC-Q-TOF (LC-MS/MS) analysis in both positive and negative ionization modes detected a comprehensive array of metabolite features across the three citrus pulp varieties. After data preprocessing and filtering, a total of [10357+] and [17198-] high-confidence metabolites were identified from all three citrus varieties. These metabolites encompassed diverse chemical classes, including flavonoids, organic acids, amino acids, sugars, and fatty acid derivatives. Quality control samples clustered tightly in multivariate models, confirming analytical reproducibility and instrument stability (Fig S1 in S1 File). The details about the metabolite profile in each citrus variety are discussed below.

#### 3.1.1. Metabolic trends in ‘Hongju 418’.

**Fig 2:**
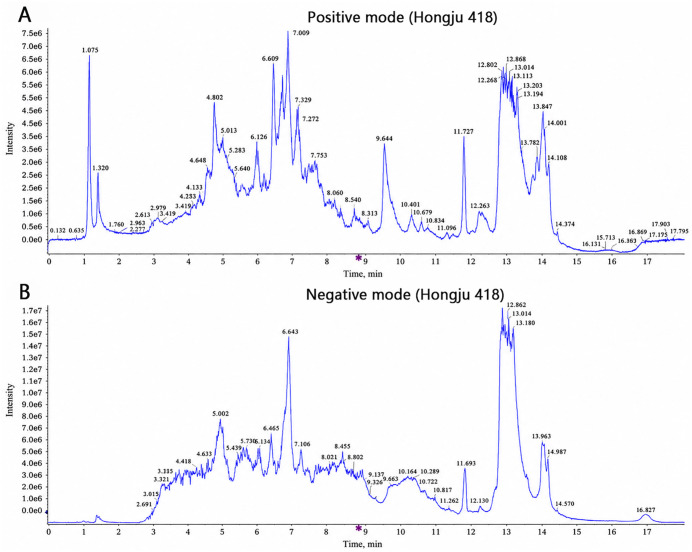
Total ion chromatogram (TIC) of Hongju 418 Tangerine Citrus variety in (A) positive ion mode and (B) negative ion mode.

The metabolomic profile of Hongju 418 revealed a distinct accumulation of specific secondary metabolite classes. Among the significantly upregulated metabolites, phenolic glycosides were the most enriched group, followed by quinic acid derivatives, coumaric acid derivatives, and iridoid O-glycosides (Fig 2; Table S1 in S1 File). These compounds are commonly associated with antioxidant properties and stress adaptation, and contribute to the typical astringent and aromatic profile of mandarins. Notably, limonoids, a class of citrus-specific triterpenoids known for their bitter taste and bioactivity, were also highly represented, suggesting a potential link to both flavor and defense responses in this variety. Additionally, 7-O-methylated flavonoids and fatty acyl hexosides were uniquely abundant, indicating metabolic specialization toward glycosylated and methylated secondary metabolites. The presence of aminoglycosides and purine nucleosides suggests an active nucleotide turnover and antimicrobial defense in Hongju 418 fruits.

#### 3.1.2. Metabolic trend in ‘Huyou’.

‘The metabolic profile of ‘Huyou’, was characterized by the significant enrichment of O-glycosyl compounds, dipeptides, and phenolic glycosides. The abundance of glycosylated secondary metabolites, including flavonoid-7-O-glycosides and flavonoid-3-O-glycosides (Fig S2; Table S1 in S1 File), suggests an extensive glycosylation pattern in Huyou, likely impacting metabolite solubility, stability, and bioavailability. The high levels of xanthines and purine nucleosides reflect dynamic purine metabolism and energy turnover, while the presence of coumaric acid derivatives and quinic acids indicates activity of the phenylpropanoid pathway activity. These features, combined with the elevated levels of 8-O-methylated flavonoids, indicate that Huyou exhibits a complex polyphenol biosynthesis network with potential antioxidant and anti-inflammatory capacity.

#### 3.1.3. Metabolic trend in ‘Xiangcheng’.

‘Xiangcheng’ exhibited a metabolome dominated by phenolic glycosides and both flavonoid-7-O- and −3-O-glycosides, consistent with roles in color, antioxidant capacity, and flavor (Fig S3; Table S1 in S1 File). O-glycosyl compounds, coumaric acid derivatives, and purine nucleosides were also prominent, indicating engagement of multiple primary and secondary pathways. Additionally, elevated flavonol O-glycosides, iridoid O-glycosides, and limonoids suggest metabolic diversification toward multiple bioactive phenolic and terpenoid groups, which likely underlie the cultivar’s flavor and nutritional profile.

### 3.2. Multivariate statistical analysis revealed clear variety-specific separation

**Fig 3:**
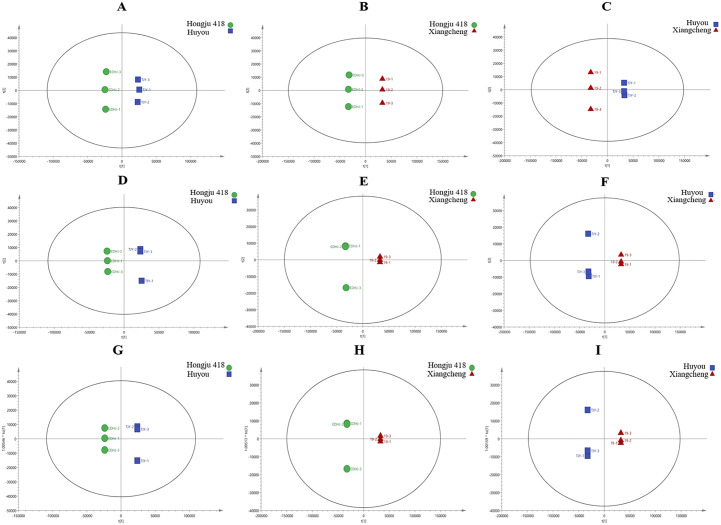
Multivariate statistical analysis of metabolite profiles across three citrus varieties—Hongju 418, Huyou, and Xiangcheng. (A–C) Principal Component Analysis (PCA) score plots showing clear within-group clustering and between-group separation in pairwise comparisons: Hongju 418 vs Huyou (A), Hongju 418 vs. Xiangcheng (B), and Huyou vs. Xiangcheng (C). (D–F) Partial Least Squares Discriminant Analysis (PLS-DA) score plots for the same comparisons, demonstrating enhanced discrimination between the varieties based on their metabolite profiles. (G–I) Orthogonal Partial Least Squares Discriminant Analysis (OPLS-DA) score plots showing refined separation and removal of irrelevant variation, further highlighting distinct metabolic patterns in each pairwise comparison.

The unsupervised PCA score plots (Fig 3A–C) revealed clear clustering patterns within each group, with distinct separation among the three citrus varieties: Hongju 418, Huyou, and Xiangcheng. The consistent within-group aggregation and between-group separation indicated good reproducibility and robust metabolomic discrimination under both positive and negative ion modes. These findings suggest that each variety possesses a unique and well-defined metabolomic fingerprint.

To further enhance group discrimination and identify differential metabolites, supervised PLS-DA models were generated (Fig 3D–F). The PLS-DA plots showed improved class separation and supported the trends observed in PCA. The corresponding R^2^ and Q^2^ values demonstrated a high degree of model fit and predictive power across all pairwise comparisons. To reduce systematic variation unrelated to class differences and minimize overfitting, OPLS-DA was applied (Fig 3G–I). The OPLS-DA plots clearly distinguished the citrus varieties based on their metabolite profiles, highlighting discriminative components that contributed most significantly to group differences. The reliability and validity of the OPLS-DA models were further confirmed through 200-iteration permutation tests (Fig S4A–C in S1 File). In all comparisons, the permuted Q^2^ values (blue dots) were substantially lower than the original Q^2^ values, which appeared on the far right of each plot. This indicates the robustness, statistical validity, and non-overfitting nature of the models. Collectively, the multivariate analysis results demonstrate that the metabolic profiles of the three citrus varieties are distinctly different and highly structured. These analyses support the identification of key biomarkers and metabolic pathways that differentiate citrus subspecies at the level of pulp tissue.

### 3.3. Identification of differential metabolites

**Fig 4:**
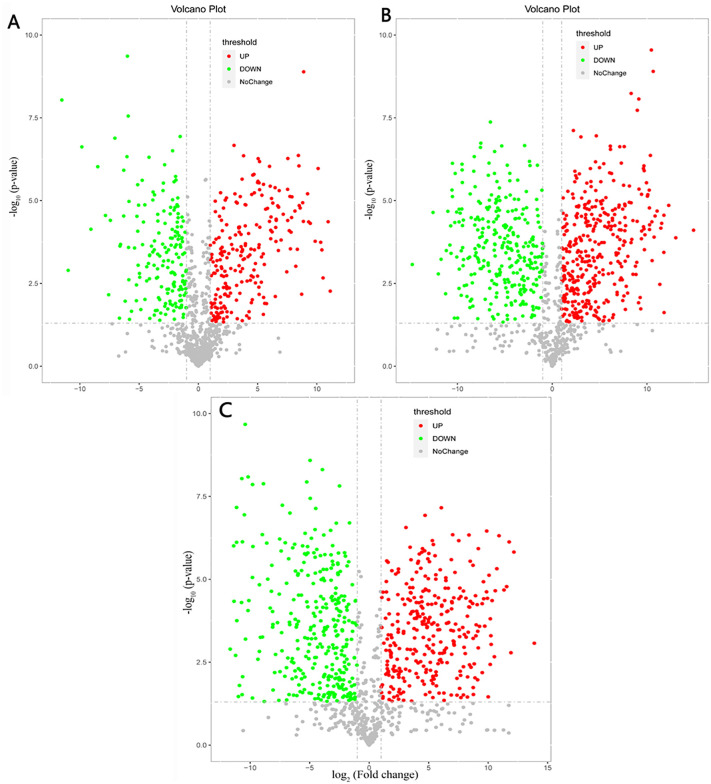
Volcano plots illustrating differentially expressed metabolites (DEMs) across citrus variety comparisons. Each volcano plot displays the distribution of DEMs based on their statistical significance (–log₁₀ (p-value), y-axis) and magnitude of change (log_2_ (Fold Change), x-axis) between the citrus groups. (A) Hongju 418 vs. Huyou (B) Hongju 418 vs. Xiangcheng (C) Huyou vs. Xiangcheng. Red dots represent metabolites significantly upregulated in the first group of each comparison; green dots indicate metabolites upregulated in the second group; and gray dots denote metabolites with no significant change. The thresholds used to define significance were VIP > 1, |log_2_ FC| ≥ 1, and p < 0.05.

Differential expressed metabolites (DEMs) were identified across the three citrus varieties using pairwise comparisons (Hongju 418 vs Huyou, Hongju 418 vs. Xiangcheng, and Huyou vs. Xiangcheng). The Hongju 418–Huyou comparison yielded roughly 280 differential metabolites, with Hongju 418 exhibiting ~165 upregulated features and Huyou ~115. More than 300 differential metabolites were identified between Hongju 418 and Xiangcheng (approximately 185 enriched in Hongju 418 and 125 in Xiangcheng). Similarly, the Huyou–Xiangcheng comparison revealed ~330 differential metabolites, including ~200 upregulated in Huyou and ~130 in Xiangcheng. These findings collectively reflect marked varietal divergence in pulp metabolism. The differential metabolites were further visualized using volcano plots (Fig 4A–C), which effectively display both the magnitude (log_2_ FC) and statistical significance (–log_10_ p-value) of metabolite changes. Red and green points represent metabolites significantly up- or downregulated, respectively, highlighting the biochemical contrast between each variety pair.

To complement the volcano plot analysis, we generated agglomerative hierarchical clustering heatmaps (Fig S5A–C in S1 File) that showcase the relative abundance patterns of the top differentially expressed metabolites across biological replicates. The heatmaps revealed clear block-wise clustering of samples by variety, with Hongju 418, Huyou, and Xiangcheng forming distinct clusters, reflecting their unique metabolomic signatures. The gradient from red to blue indicates metabolite enrichment and depletion, respectively, across the groups. Notably, phenolic glycosides, flavonoid O-glycosides, limonoids, and quinic acid derivatives were frequently represented among the top DEMs, suggesting their potential as chemotaxonomic markers. These compounds are known to influence the taste, color, nutritional quality, and bioactivity of citrus pulp. The direction and magnitude of their changes across pairwise comparisons further support their roles in varietal differentiation.

### 3.4. Correlation analysis among differential metabolites

Pearson correlation heatmaps for each comparison are presented in Fig S6A–C in S1 File. These heatmaps employ red and blue gradients to represent positive and negative correlations, respectively, and hierarchical clustering to delineate metabolite modules with shared expression patterns. To investigate co-regulatory relationships among differentially expressed metabolites, Pearson correlation coefficient (PCC) analysis was performed for each pairwise comparison—Hongju 418 vs Huyou, Hongju 418 vs Xiangcheng, and Huyou vs Xiangcheng. This analysis enabled the identification of tightly co-varying metabolites, thereby revealing functional metabolic modules and shared biosynthetic or regulatory pathways. In the Hongju 418 vs Huyou comparison, strong positive correlations (|R| ≥ 0.8) were predominantly found among flavonoid O-glycosides, methylated flavonoids, and limonoids, which were enriched in Hongju 418. These metabolites formed cohesive clusters reflecting a highly coordinated flavonoid biosynthetic network. In contrast, Huyou displayed stronger intra-group correlations among organic acid derivatives, iridoid glycosides, and xanthine-based alkaloids, suggesting enhanced primary carbon flux and terpene-related metabolism. A similar trend was observed in the Hongju 418 vs Xiangcheng comparison, where Hongju 418 again showed coordinated accumulation of flavonoid-related metabolites. Meanwhile, Xiangcheng exhibited distinct correlation modules comprising amino acid conjugates, carboxylic acids, and sugar alcohols, consistent with a metabolic program geared towards energy homeostasis and primary metabolite regulation.

In the Huyou vs Xiangcheng comparison, Huyou showed robust correlations within limonoid aglycones, fatty acyl hexosides, and phenolic glycosides, indicating extensive diversification of secondary metabolites. Conversely, Xiangcheng maintained coherent associations among flavonoid-7-O-glycosides and malic acid derivatives, highlighting its characteristic metabolic orientation. Collectively, the results reveal variety-specific metabolic co-regulation, with clearly defined clusters emerging across comparisons. Such modular behavior underscores genotype-driven organization of citrus pulp metabolism and identifies potential biosynthetic nodes relevant to flavor and nutritional traits.

### 3.5. KEGG pathway enrichment highlights variety-specific metabolic pathways

**Fig 5:**
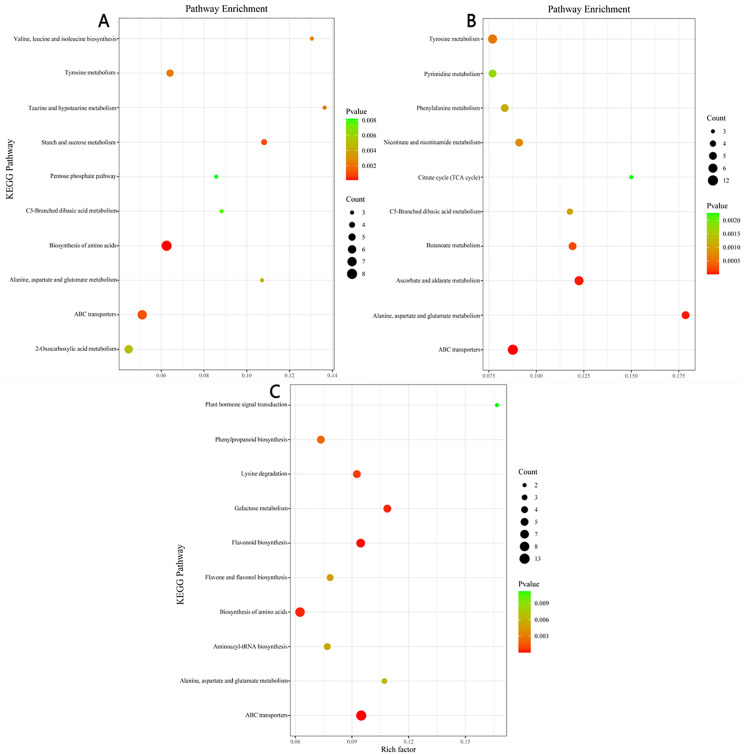
KEGG pathway enrichment analysis of differentially expressed metabolites (DEMs) in pairwise comparisons among three citrus varieties. Differential metabolites (VIP > 1, |log_2_FC| ≥ 1, p < 0.05) were mapped to KEGG pathways to assess their functional relevance using enrichment statistics. (A) KEGG bubble chart showing enriched metabolic pathways in the Hongju 418 vs Huyou comparison. (B) KEGG enrichment results for Hongju 418 vs Xiangcheng, (C) KEGG bubble plots for Huyou vs Xiangcheng. In bubble plots, the x-axis represents the rich factor (proportion of DEMs in a given pathway), the y-axis lists the pathway names, bubble size corresponds to the number of metabolites mapped, and color gradient indicates adjusted p-values (darker colors = more significant enrichment).

To clarify the biological relevance of DEMs, KEGG pathway enrichment analysis was performed for each pairwise comparison: Hongju 418 vs. Huyou, Hongju 418 vs. Xiangcheng, and Huyou vs. Xiangcheng. DEMs were mapped to metabolic pathways in the Kyoto Encyclopedia of Genes and Genomes (KEGG) database, and enrichment significance was assessed using the rich factor, adjusted p-values, and pathway hit numbers. In the Hongju 418 vs. Huyou comparison, DEMs were significantly enriched in biosynthesis of amino acids, ABC transporters, tyrosine metabolism, pentose phosphate pathway, and starch and sucrose metabolism (Fig 5A). These pathways indicate pronounced divergence in core nitrogen metabolism, carbohydrate partitioning, and energy metabolism between the two genotypes. The enrichment of amino acid biosynthesis and the pentose phosphate pathway is particularly noteworthy, suggesting differential metabolic allocation toward nitrogen assimilation and reducing power generation between the tangerine genotype and the hybrid.

For the Hongju 418 vs. Xiangcheng comparison, DEMs were primarily enriched in the citrate cycle (TCA cycle), ascorbate and aldarate metabolism, phenylalanine metabolism, tyrosine metabolism, and alanine, aspartate and glutamate metabolism (Fig 5B). These pathways align with the high abundance of organic acids, antioxidants, and amino acid-related metabolites in Xiangcheng, underscoring its metabolic allocation toward core energy metabolism, stress defense and aromatic compound formation. The enrichment of the TCA cycle and ascorbate metabolism further supports the divergence in respiratory carbon flux and antioxidant capacity between the two varieties. In the Huyou vs. Xiangcheng comparison, phenylpropanoid biosynthesis, flavonoid biosynthesis, flavone and flavonol biosynthesis, and plant hormone signal transduction were significantly enriched (Fig 5C). The enrichment reflects the pronounced accumulation of phenolic and flavonoid derivatives in the respective genotypes, highlighting metabolic features associated with secondary metabolite biosynthesis and specialized defense responses. In contrast, the co-enrichment of amino acid biosynthesis and ABC transporters indicates a shared metabolic tendency toward nitrogen assimilation and metabolite transport between the two varieties.

### 3.6. Key metabolite markers discriminating citrus varieties

Several metabolites exhibited strong discriminatory power among citrus subspecies. Naringenin and hesperidin were markedly enriched in Hongju 418; succinic acid and caffeic acid derivatives were elevated in Huyou; and citric acid, D-glucaric acid, and malic acid were more abundant in Xiangcheng. These patterns demonstrate substantial divergence in pulp metabolic composition and highlight their potential utility for quality assessment and functional food development.

## 4. Discussion

Studying the diversity of metabolites among different varieties of citrus fruits is essential for the healthy development of the citrus industry. This study employed a high-resolution, untargeted LC-MS/MS-based metabolomics approach to analyze the metabolic diversity of the pulp of three citrus varieties—Hongju 418, Huyou, and Xiangcheng. Our findings reveal significant metabolomic divergence, particularly in flavonoids, limonoids, organic acids, and terpenoids, providing unprecedented insight into the biochemical differentiation among different citrus genotypes.

### 4.1. Genotype-driven chemotype divergence in citrus pulps

The clear separation of samples across PCA, PLS-DA, and OPLS-DA models demonstrates that genetic factors strongly regulate citrus pulp metabolomes. This distinct clustering confirms that citrus chemotypes are shaped not only by external factors but also by stable, genotype-encoded metabolic configurations. While previous studies have emphasized peel volatiles and flavonoids [34–37], our results extend this understanding to pulp tissues, revealing that even genetically related cultivars, such as Hongju 418 and Huyou, harbor markedly different metabolite profiles. Divergence in flavonoid glycosides, polymethoxylated flavonoids, limonoid aglycones, and terpenoid derivatives primarily defines the functional chemotypic identity of each pulp, directly influencing sensory perception and bioactive potential [38,39]. The unique metabolic profile of the ancestral genotype Xiangcheng suggests that metabolite signatures may reflect phylogenetic trajectories, domestication processes, and adaptive responses. Notably, the three genotypes diverge not merely in metabolite abundance, but in the hierarchical architecture of their secondary metabolite classes: Hongju 418 specializes in flavonoid O-glycosylation and methylation, Xiangcheng accumulates terpenoid and limonoid scaffolds alongside phenolic acids, and Huyou exhibits hybrid-specific transgressive segregation in caffeoyl-quinic acid conjugates and xanthine alkaloids. These class-level distinctions form the basis for genotype-specific functional food applications. These findings demonstrate that pulp metabolomics can serve as a robust platform for germplasm discrimination and for guiding breeding efforts targeting specialized metabolite enrichment and functional quality.

### 4.2. Flavonoid-rich signature in Hongju 418 citrus variety

One of the most notable outcomes of our metabolomic analysis is the pronounced enrichment of flavonoid compounds in the pulp of Hongju 418. Specifically, subclasses such as flavonoid O-glycosides, 7-O-methylated flavonoids, and flavonol derivatives were significantly over-accumulated and exhibited high intra-cluster correlations, indicating coordinated biosynthetic regulation. This chemotypic trait aligns with previous observations in *Citrus reticulata* species, which are renowned for its rich flavonoid profile, particularly in peel tissues [40]. However, our study is among the few to report such consistent flavonoid abundance in pulp tissue, which is directly relevant to dietary intake. The co-accumulation pattern observed suggests a transcriptionally coordinated mechanism likely governed by R2R3-MYB and bHLH transcription factors, as supported by studies in citrus callus and fruit peel systems [3,41]. These regulatory modules are known to activate structural genes such as CHS, FNS, and UGTs, catalyzing key steps in flavonoid biosynthesis and glycosylation. For instance, the accumulation of hesperidin, naringenin-7-O-glucoside, and other glycosylated derivatives highlighted in our dataset has been linked to health-promoting properties, including antioxidant, neuroprotective, cardioprotective, and anti-inflammatory activities [42,43]. Moreover, the specific overrepresentation of 7-O-methylated flavonoids, which are relatively uncommon and often associated with higher metabolic stability and bioavailability, adds further nutraceutical significance to Hongju 418 [44]. Comparative analysis further revealed that Hongju 418 pulp is uniquely enriched in diosmetin-7-O-rutinoside and narirutin-4’-O-glucoside, which were negligible in Huyou and Xiangcheng. The positional specificity of O-glycosylation (7-O- vs. 4’-O-) and the high methoxylation ratio suggest a refined post-biosynthetic modification capacity in this cultivar, potentially enhancing intestinal absorption and metabolic stability [45]. This cultivar thus represents a specialized flavonoid chemotype, offering a genetic resource for breeding programs targeting O-glycosylated and O-methylated flavonoid enrichment in fresh pulp. These findings not only validate the nutritional potential of *Citrus reticulata* derivatives but also underscore the importance of targeted metabolomics in guiding cultivar selection for health-beneficial traits.

### 4.3. Limonoid- and phenolic-enriched secondary metabolome in Xiangcheng citrus variety

While Xiangcheng maintains elevated primary organic acids that define its characteristic tartness [21,46,47], its pulp is concurrently distinguished by a terpenoid- and limonoid-enriched secondary metabolome. Xiangcheng pulp exhibited significant accumulation of limonin glucoside, nomilinic acid, and obacunone derivatives—limonoid aglycones and glucosides that are scarce in Hongju 418 and moderate in Huyou. These triterpenoid secondary metabolites, derived from the mevalonate and methylerythritol phosphate pathways, confer pronounced bitter-masking potential and anti-carcinogenic activity [48]. Additionally, Xiangcheng was uniquely enriched in hydroxycinnamate conjugates, particularly feruloyl- and sinapoyl-quinic acids, which were barely detectable in the other two varieties. The co-expression of these phenolic esters with terpenoid scaffolds suggests a coordinated upregulation of the phenylpropanoid-isoprenoid cross-talk, a metabolic interface often associated with enhanced oxidative stress tolerance [49]. This secondary metabolite configuration positions Xiangcheng as a candidate for limonoid-based nutraceutical extraction, distinct from the flavonoid-oriented profile of Hongju 418

### 4.4. Hybrid metabolic complexity in Huyou citrus variety

Huyou presented a distinct secondary metabolite landscape consistent with its hybrid origin, displaying transgressive accumulation of caffeic acid hexosides, theobromine, and fatty acyl conjugates that exceeded both parental chemotypes. Notably, Huyou pulp accumulated novel hybrid-specific metabolites, including 3,5-di-O-caffeoylquinic acid and 1-O-sinapoyl-β-glucose, which were absent or trace in the parental genotypes. These phenolic conjugates likely arise from the combinatorial expression of parental UDP-glycosyltransferase (UGT) and hydroxycinnamoyl-CoA: quinate transferase (HQT) alleles, illustrating how genomic hybridity expands the biochemical repertoire of specialized metabolism [50]. The co-accumulation of xanthine alkaloids (theobromine, caffeine) and caffeoyl-quinic acids further suggests an integrated defense-oriented chemotype, combining antimicrobial phenolics with nitrogen-containing secondary metabolites [51]. This metabolic plasticity in specialized metabolism exemplifies how interspecific hybridization can generate non-additive secondary metabolite traits for functional food innovation. These findings provide system-level biochemical validation of Huyou's hybrid nature, underscoring the utility of metabolomics in unravelling complex genotype-phenotype interactions and guiding the exploitation of hybrid vigor in citrus breeding.

### 4.5. Pathway and network-level integration of metabolic signatures

The integrated metabolomic framework employed in this study—combining correlation profiling, module detection, and KEGG pathway enrichment—unveils that each citrus genotype channels its secondary metabolic activity along distinct functional and evolutionary trajectories. These genotype-specific configurations are not merely biochemical footprints; they reflect deep genetic encoding of specialized metabolism, ecophysiological adaptation, and domestication legacies [51,52]. In Hongju 418, the metabolic network is prominently structured around a flavonoid biosynthesis module, with tight co-expression among CHS, FNS, and UGT downstream products (hesperidin, naringenin-7-O-glucoside, and diosmetin derivatives), indicating canalized selection for flavonoid glycosylation and methylation [53,54]. The modular clustering of these compounds suggests a high degree of transcriptional co-regulation, possibly reflecting canalized selection for the production of bioactive metabolites. Xiangcheng exhibited a distinct ‘limonoid-terpenoid’ network module, wherein limonin glucoside, nomilinic acid, and obacunone showed strong intra-module correlation, coupled to feruloyl-quinic acids via the phenylpropanoid interface. This module likely reflects the activation of the mevalonate and phenylpropanoid pathways under genotype-specific transcriptional control [49]. Huyou, by contrast, displayed a ‘hybrid phenolic-alkaloid’ module characterized by the co-regulation of di-O-caffeoylquinic acids and xanthines, suggesting a unique transcriptional synergy between the phenylpropanoid and purine alkaloid pathways that is absent in the parental genotypes [55]. This hybrid-specific secondary metabolite integration supports metabolic plasticity in specialized metabolism, traits commonly associated with heterosis and hybrid vigor in perennial crops [56,57].

These genotype-specific secondary metabolite modules are not merely biochemical footprints; they reflect deep genetic encoding of specialized metabolism, shaped by evolutionary lineage and human selection. This study provides robust system-level evidence that secondary metabolite diversity in citrus pulp is heritable, modular, and predictive, thereby opening avenues for precision breeding through metabolite-guided selection targeting flavonoid, limonoid, or phenolic acid enrichment, and for the enhancement of nutritional and functional profiles in citrus-derived foods.

## 5. Conclusions

This study delivers a comprehensive metabolomic comparison of three citrus pulp varieties using non-targeted LC-MS/MS profiling. Our results highlight pronounced metabolic distinctions among *Citrus reticulata* ‘Hongju 418’, *Citrus aurantium* ‘Changshan-huyou’ and *Citrus junos* ‘Hunan Xiangcheng’, reflecting both species lineage and hybridization effects. The identification of distinct metabolic signatures—enriched in flavonoids, organic acids, and lipophilic compounds—provides functional insights into citrus quality traits. Moreover, integrating correlation analysis with KEGG pathway enrichment reveals genotype-specific biosynthetic regulation. These findings serve as a foundational reference for future citrus breeding, functional evaluation, and targeted metabolite engineering.

## Supporting information

S1 File Appendix Figure and Table in this study. This file includes individual sheets for each assay: total ion chromatogram, validation of the OPLS-DA models, hierarchical clustering heatmaps of differentially expressed metabolites, Pearson correlation heatmaps, and representative differentially expressed metabolites.
(PDF)

S1 Data Data. (ZIP)
