# Supplementary material for: Preliminary study on comparative non-targeted metabolomics analysis sheds light on the chemical diversity of citrus fruit pulps
Source: PLoS One. 2026 Jul 22;21(7):e0353350. doi: 10.1371/journal.pone.0353350 (PMC13390813; doi:10.1371/journal.pone.0353350)
Supplement: S1 File — This file includes individual sheets for each assay: total ion chromatogram, validation of the OPLS-DA models, hierarchical clustering heatmaps of differentially expressed metabolites, Pearson correlation heatmaps, and representative differentially expressed metabolites. (PDF) [file pone.0353350.s001.pdf]

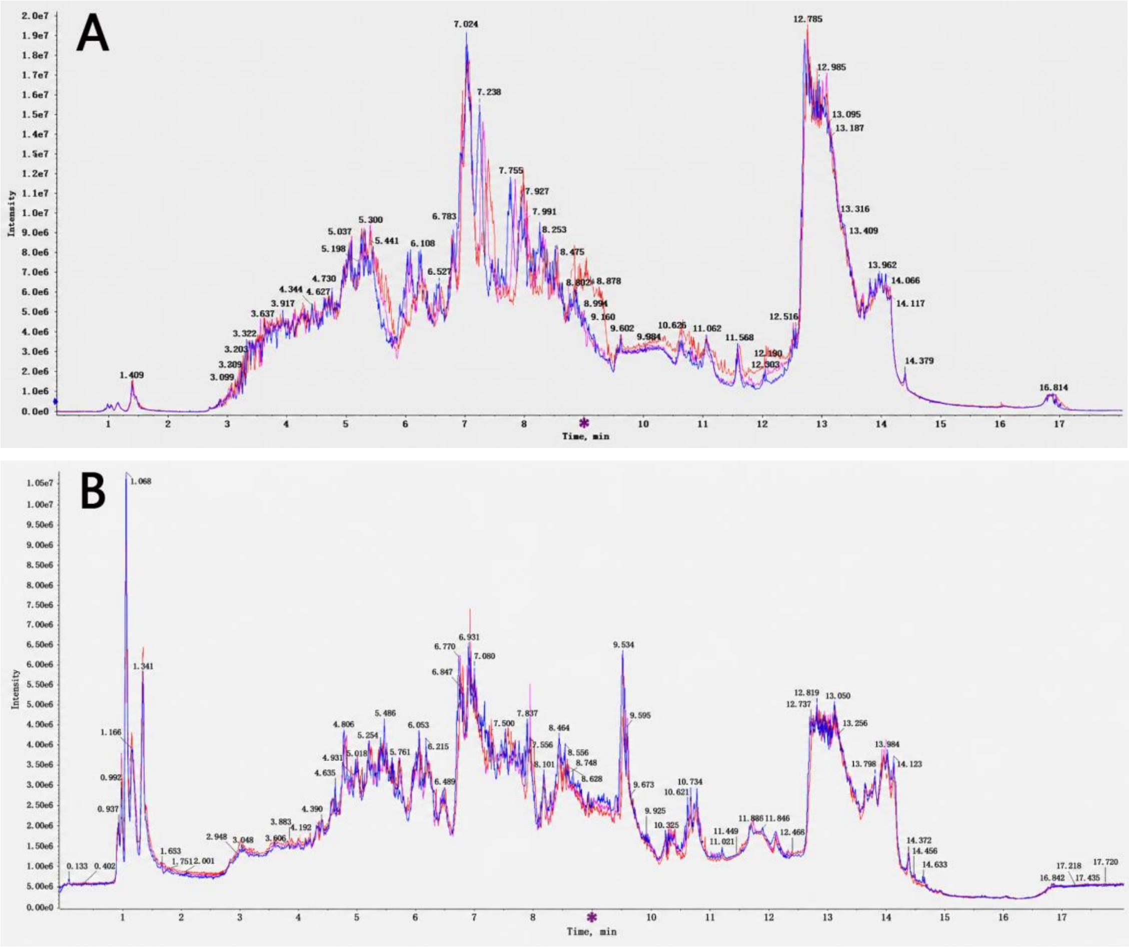

Fig S1. Overlaid total ion chromatograms (TICs) of quality control (QC) samples acquired in negative-ion mode(A) and positive-ion mode(B).

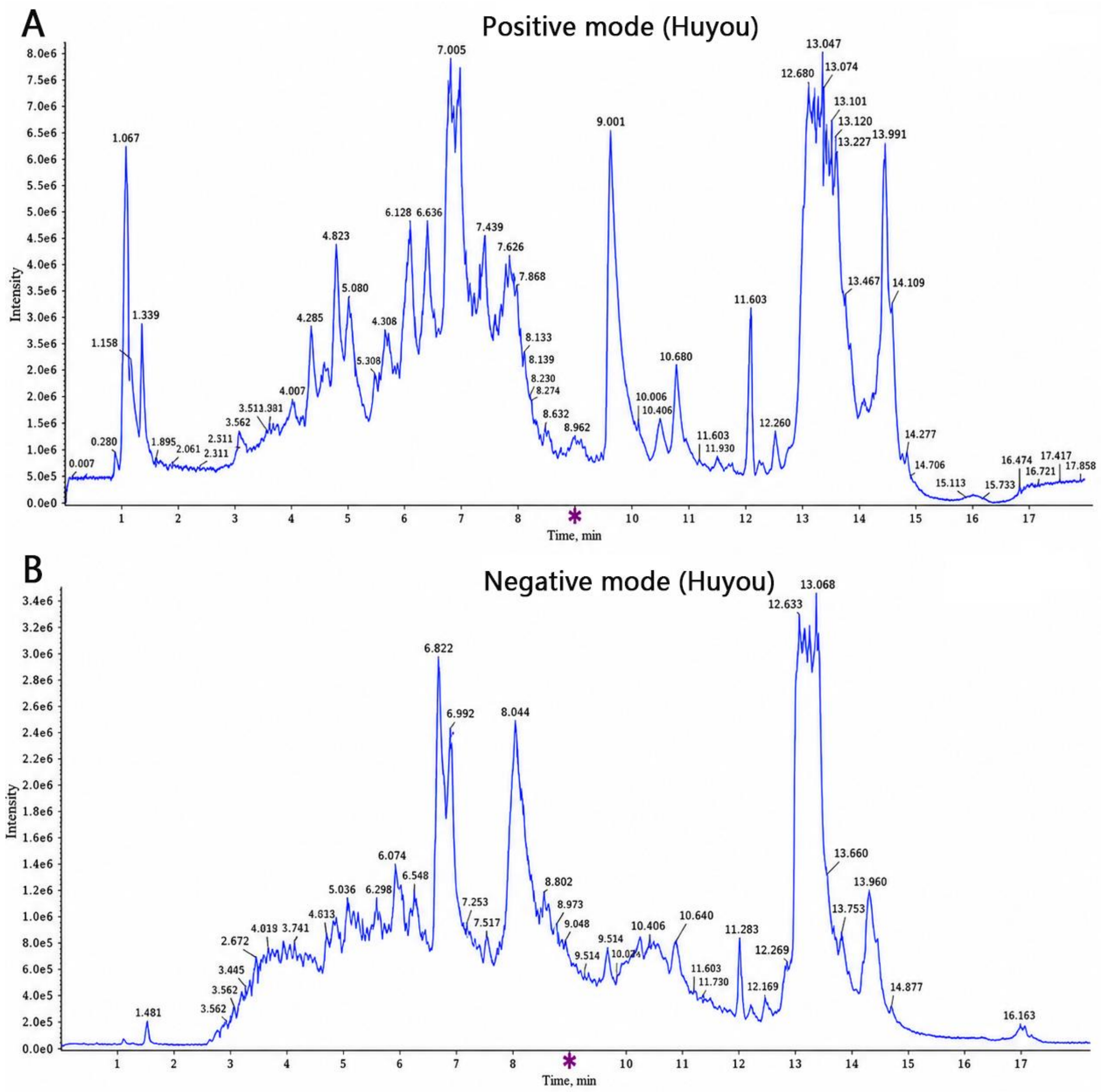

Fig S2. Total ion chromatogram (TIC) of Huyou Pomelo Citrus variety in (A) positive ion mode and (B) negative ion mode.

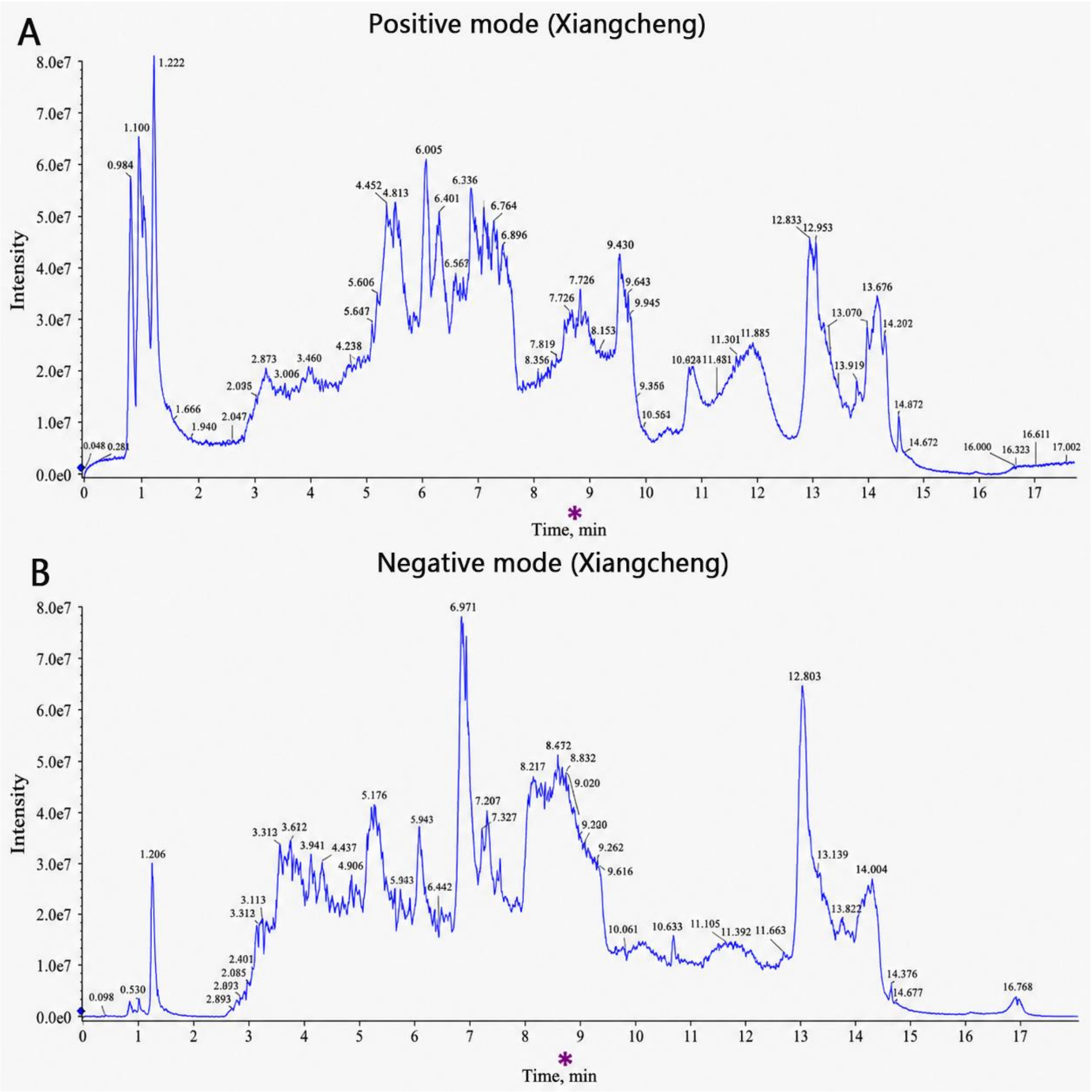

Fig S3. Total ion chromatogram (TIC) of *Xiangcheng* Orange Citrus variety in (A) positive ion mode and (B) negative ion mode.

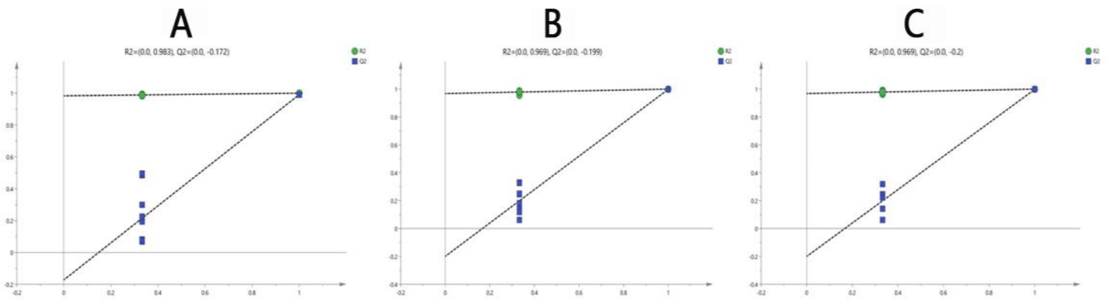

Fig S4. (A–C) Permutation test validation of the OPLS-DA models (200 iterations), illustrating that the original Q^2 values (black dots on the right) are significantly higher than those of the permuted models (blue dots), confirming that the models are robust, predictive, and not overfitted.

A

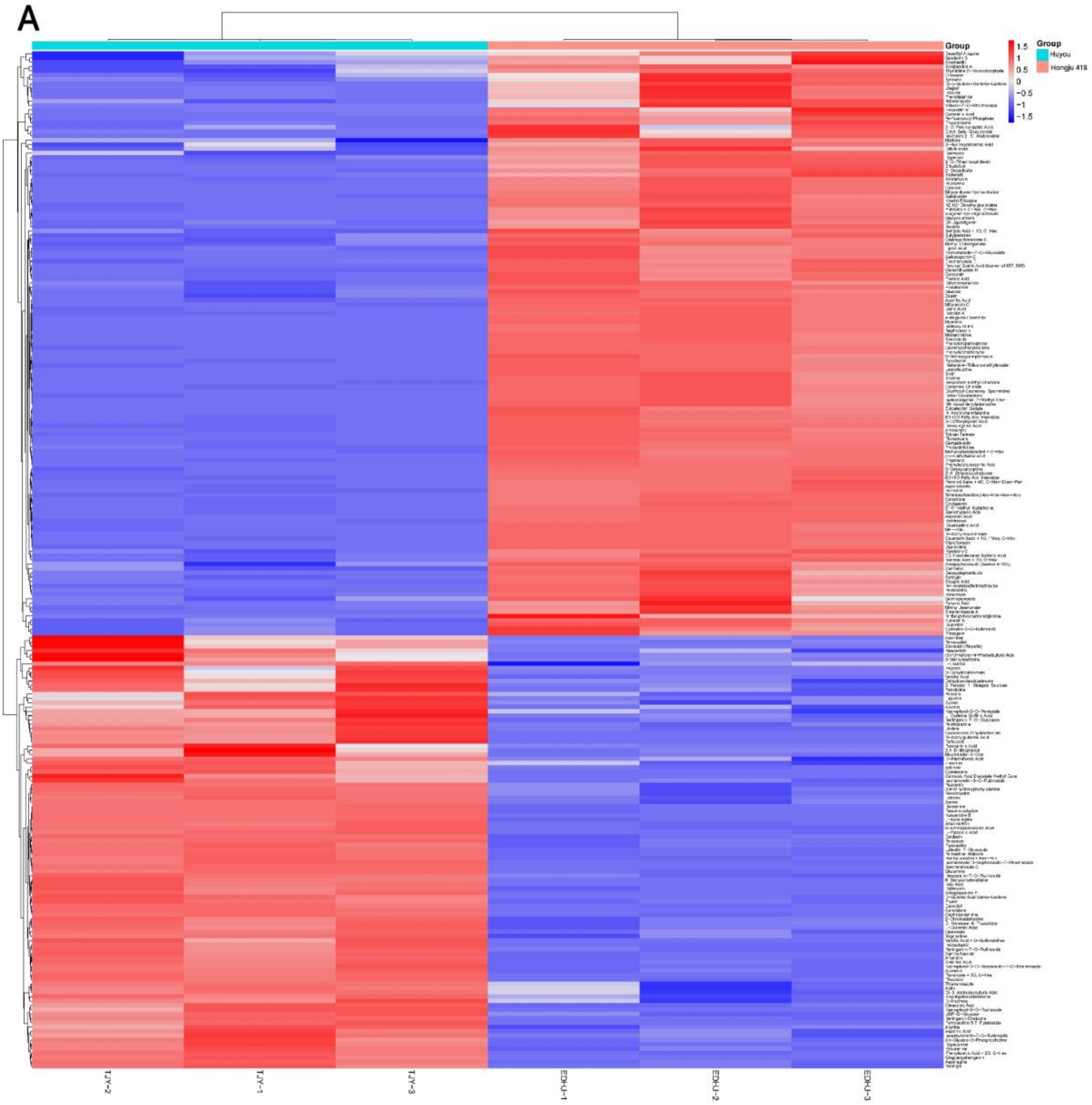

Fig S5A. Hierarchical clustering heatmaps of differentially expressed metabolites (DEMs) across two citrus varieties (Hongju 418 vs. Huyou). Rows represent individual DEMs and columns represent biological replicates. The color gradient from blue to red indicates the z-score normalized relative abundance, with red and blue denoting high and low abundance levels, respectively. The top-colored bars indicate the variety identity of each sample. DEMs were selected based on VIP > 1, $|\log_2 FC| \geq 1$, and $p < 0.05$, as shown in the volcano plots (Fig 4A).

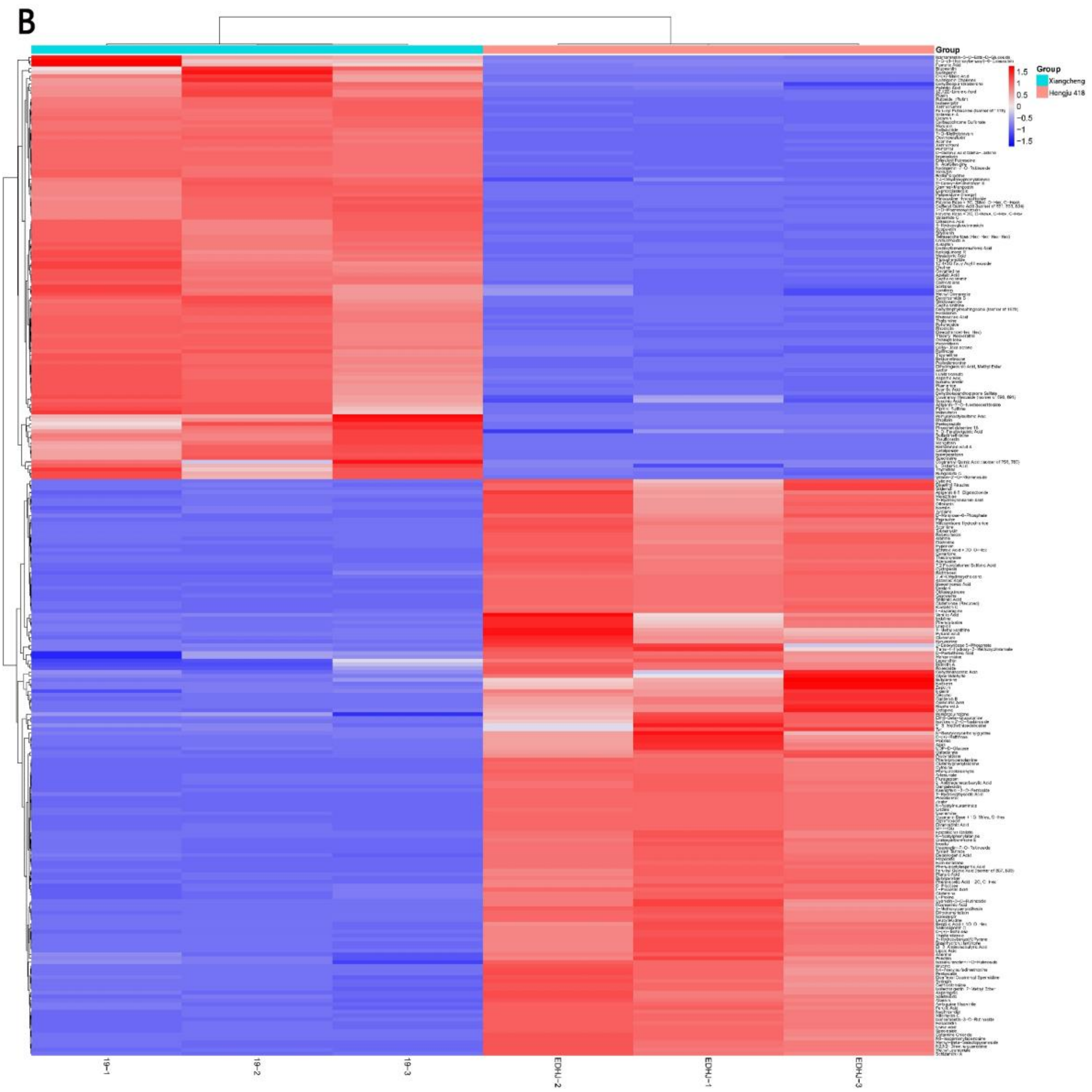

Fig S5B. Hierarchical clustering heatmaps of differentially expressed metabolites (DEMs) across two citrus varieties (Hongju 418 vs. Xiangcheng). Rows represent individual DEMs and columns represent biological replicates. The color gradient from blue to red indicates the z-score normalized relative abundance, with red and blue denoting high and low abundance levels, respectively. The top-colored bars indicate the variety identity of each sample. DEMs were selected based on VIP > 1, $|\log_2 FC| \geq 1$, and $p < 0.05$, as shown in the volcano plots (Fig 4B).

C

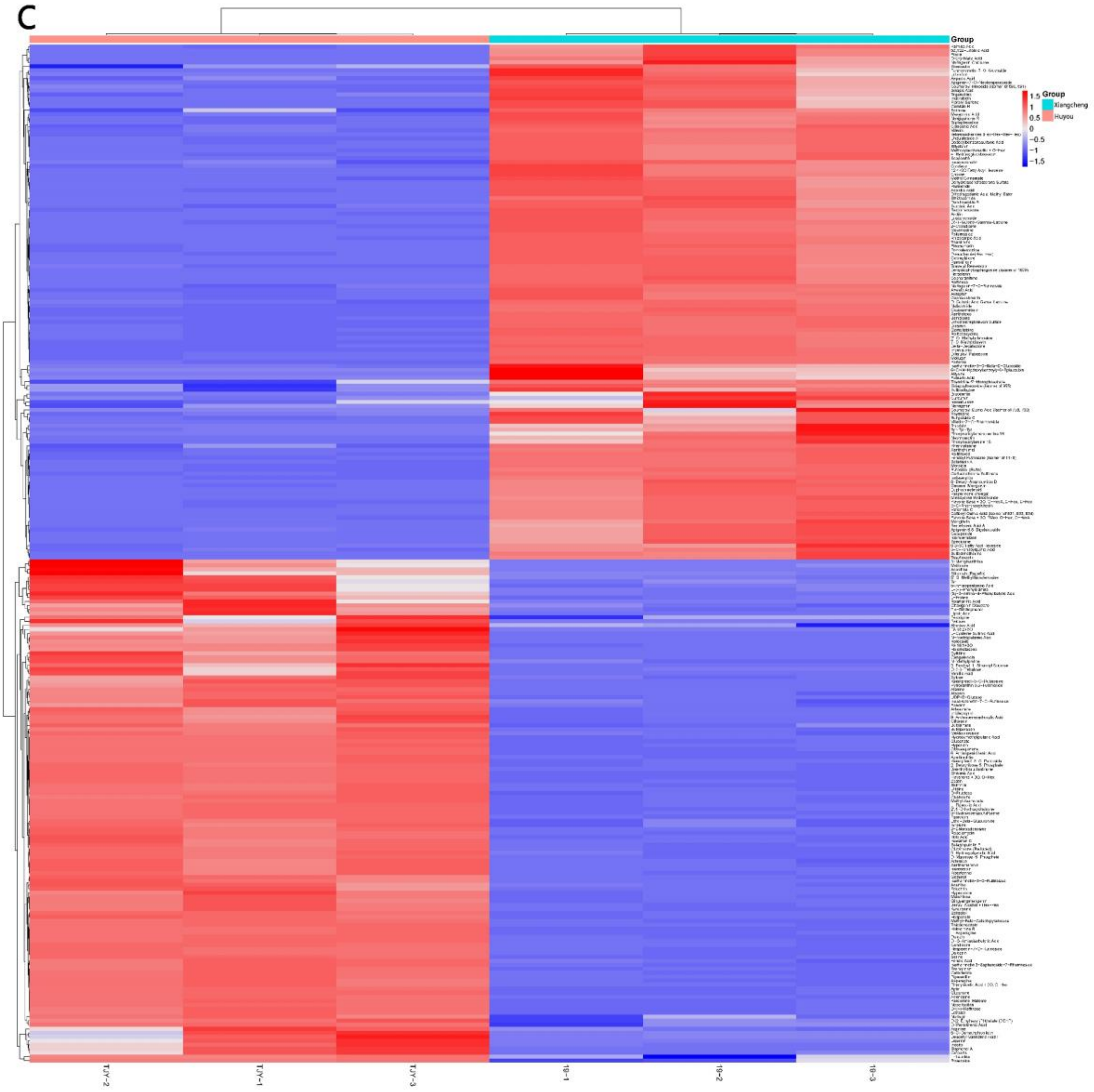

Fig S5C. Hierarchical clustering heatmaps of differentially expressed metabolites (DEMs) across two citrus varieties (Huyou vs. Xiangcheng). Rows represent individual DEMs and columns represent biological replicates. The color gradient from blue to red indicates the z-score normalized relative abundance, with red and blue denoting high and low abundance levels, respectively. The top-colored bars indicate the variety identity of each sample. DEMs were selected based on VIP > 1, $|\log_2 FC| \geq 1$, and $p < 0.05$, as shown in the volcano plots (Fig 4C).

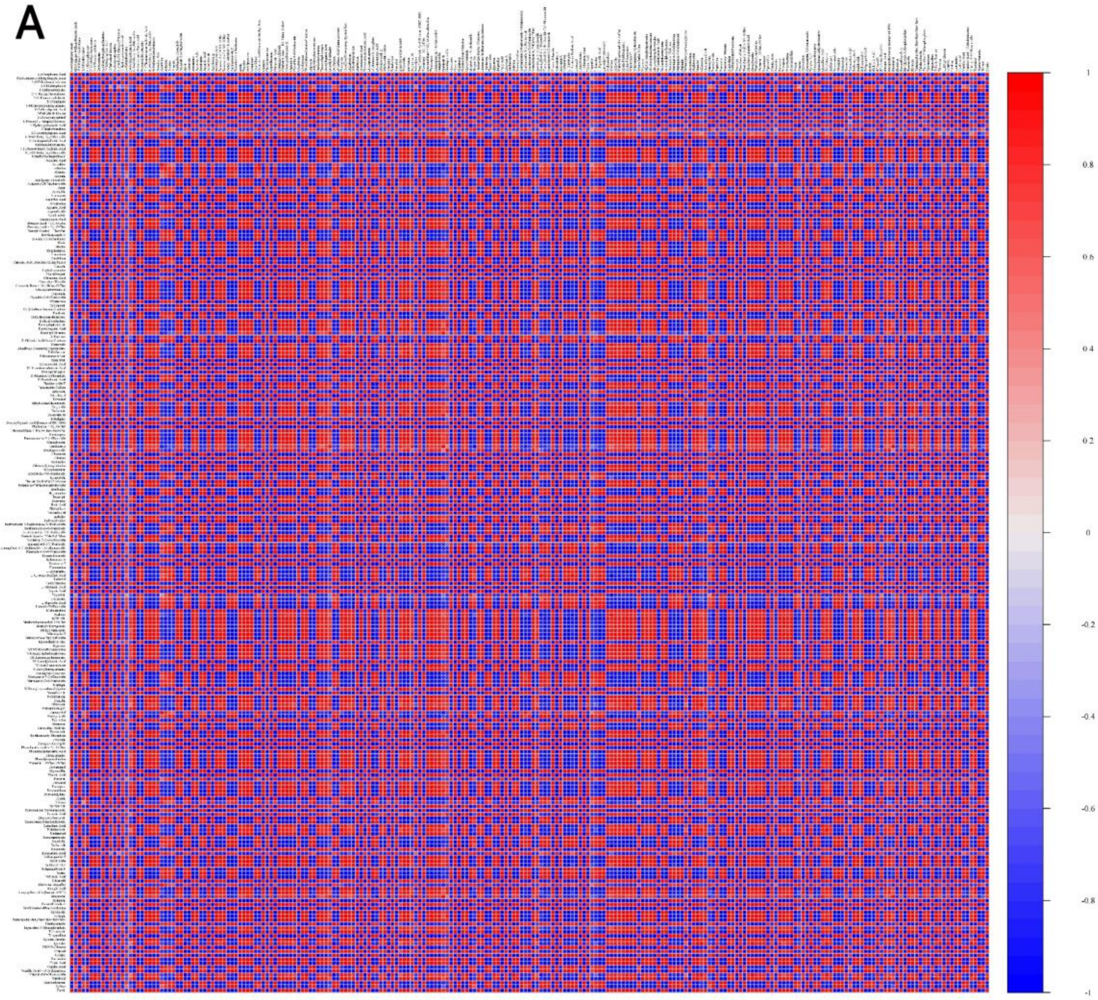

Fig S6A. Pearson correlation heatmaps of differentially expressed metabolites (DEMs) across pairwise comparisons of citrus varieties (Hongju 418 vs. Huyou). Hierarchical clustering was applied to both rows and columns to group metabolites with shared correlation patterns. The color gradient from blue to red represents the Pearson correlation coefficient (r), ranging from -1 (strong negative correlation) through 0 (no correlation) to +1 (strong positive correlation). DEMs were identified based on VIP > 1, $|log_2FC| \geq 1$, and $p < 0.05$.

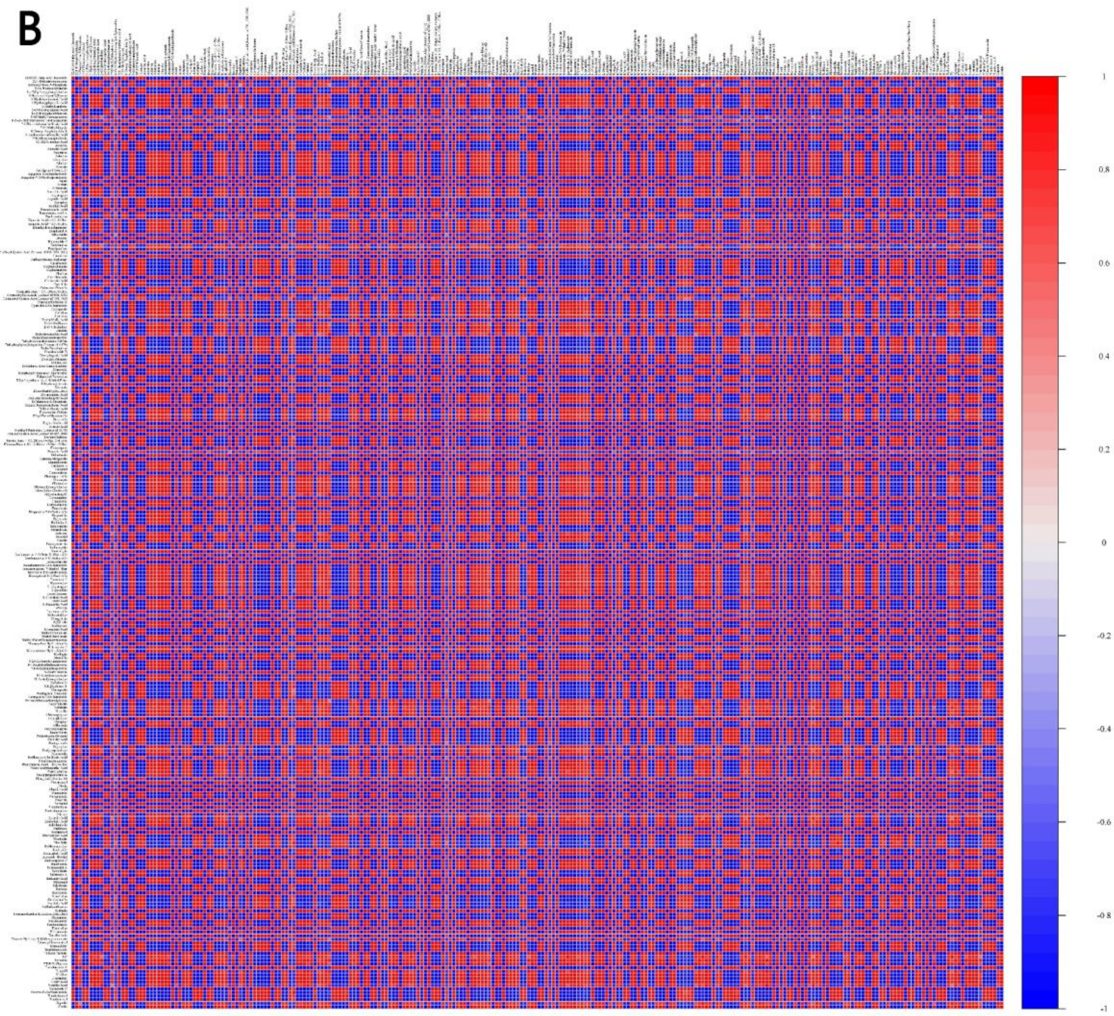

Fig S6B. Pearson correlation heatmaps of differentially expressed metabolites (DEMs) across pairwise comparisons of citrus varieties (Hongju 418 vs. Xiangcheng). Hierarchical clustering was applied to both rows and columns to group metabolites with shared correlation patterns. The color gradient from blue to red represents the Pearson correlation coefficient (r), ranging from -1 (strong negative correlation) through 0 (no correlation) to +1 (strong positive correlation). DEMs were identified based on VIP > 1, $|\log_2 FC| \geq 1$, and $p < 0.05$.

C

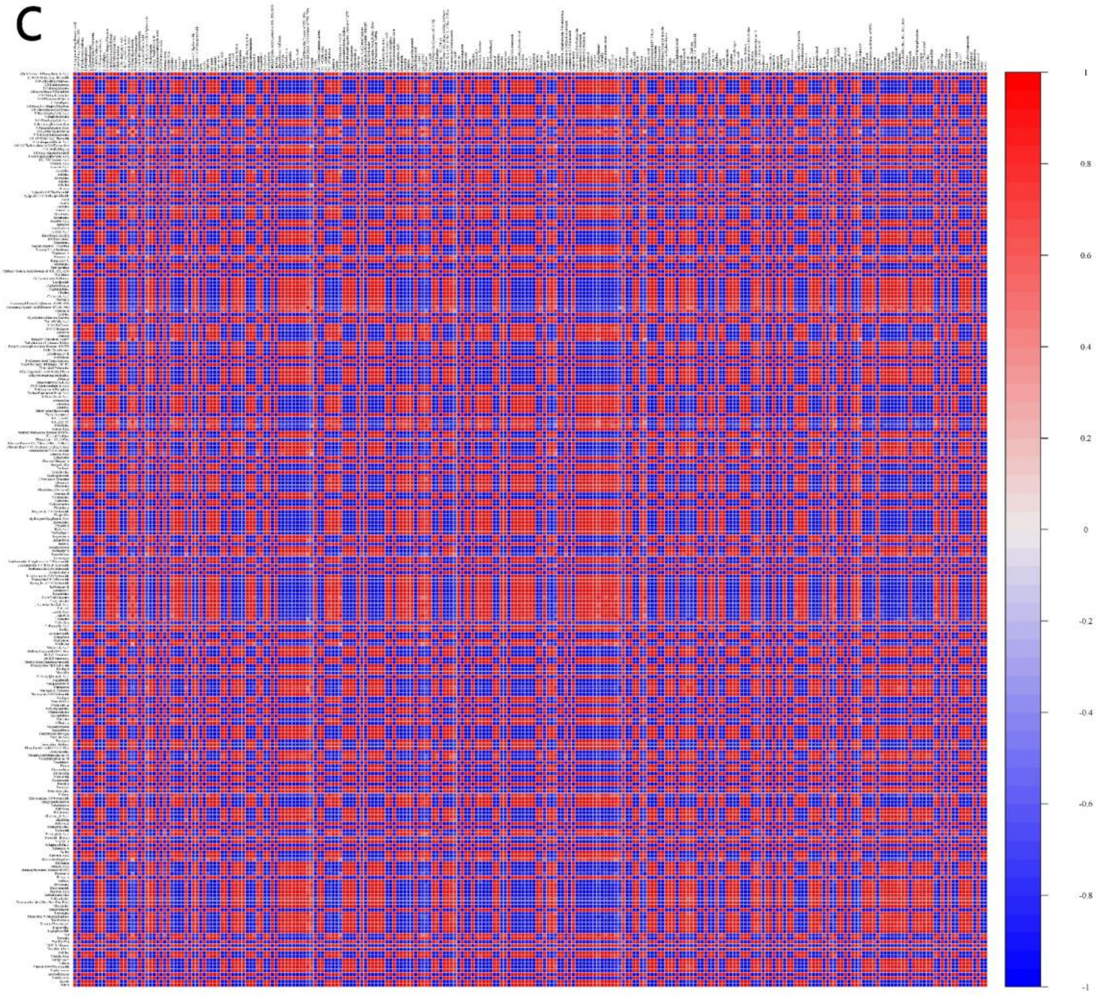

Fig S6C. Pearson correlation heatmaps of differentially expressed metabolites (DEMs) across pairwise comparisons of citrus varieties (Huyou vs. Xiangcheng). Hierarchical clustering was applied to both rows and columns to group metabolites with shared correlation patterns. The color gradient from blue to red represents the Pearson correlation coefficient (r), ranging from -1 (strong negative correlation) through 0 (no correlation) to +1 (strong positive correlation). DEMs were identified based on VIP > 1, $|\log_2 FC| \geq 1$, and $p < 0.05$.

Table S1. Representative differentially expressed metabolites across three citrus varieties.

Metabolite	Chemical class	RT (min)	m/z	Mode	Log_2FC (HJ vs HY)	Log_2FC (HJ vs XC)	Log_2FC (HY vs XC)	*p*-value	Enriched variety
Quercetin-3-O-glucoside	Flavonoid glycoside	8.52	463.088	Neg	3.42	2.89	−0.53	<0.001	Hongju 418
Limonin	Limonoid	12.45	469.186	Neg	4.15	3.68	−0.47	<0.001	Hongju 418
Chlorogenic acid	Quinic acid derivative	5.85	353.088	Neg	2.75	2.31	−0.44	0.002	Hongju 418
7-O-Methylated flavonoid	Flavonoid	9.12	299.056	Neg	1.85	1.52	−0.33	0.008	Hongju 418
Esculin	Phenolic glycoside	6.95	341.088	Pos	2.56	2.12	−0.44	0.003	Hongju 418
Naringin	Flavonoid glycoside	8.78	579.171	Neg	−3.05	−2.67	0.38	<0.001	Huyou
Rutin	Flavonoid glycoside	9.15	609.146	Neg	−2.81	−2.45	0.36	<0.001	Huyou
Xanthine	Purine alkaloid	4.15	153.041	Pos	−1.82	−1.45	0.37	0.008	Huyou
Leu-Ile	Dipeptide	3.56	245.186	Pos	−1.65	−1.28	0.37	0.015	Huyou
Hesperidin	Flavonoid glycoside	10.15	609.182	Neg	2.41	−1.93	−4.34	<0.001	Xiangcheng

Quercetin-3-O-rhamnoside	Flavonol glycoside	8.95	447.093	Neg	1.62	−2.15	−3.77	0.003	Xiangcheng
Nomilin	Limonoid	11.85	483.248	Pos	−1.25	−3.68	−4.93	<0.001	Xiangcheng
Secologanin	Iridoid glycoside	7.65	391.114	Pos	−1.85	−2.45	−4.30	0.005	Xiangcheng

Note: *HJ = Hongju 418; HY = Huyou; XC = Xiangcheng. Log_2FC positive values indicate upregulation in the first group of each comparison. Identification was based on MS/MS spectral matching against HMDB and MassBank (tolerance < 25 ppm).*
