# Supplementary figures and images for: Preliminary study on comparative non-targeted metabolomics analysis sheds light on the chemical diversity of citrus fruit pulps
Source: PLoS One. 2026 Jul 22;21(7):e0353350. doi: 10.1371/journal.pone.0353350 (PMC13390813; doi:10.1371/journal.pone.0353350)

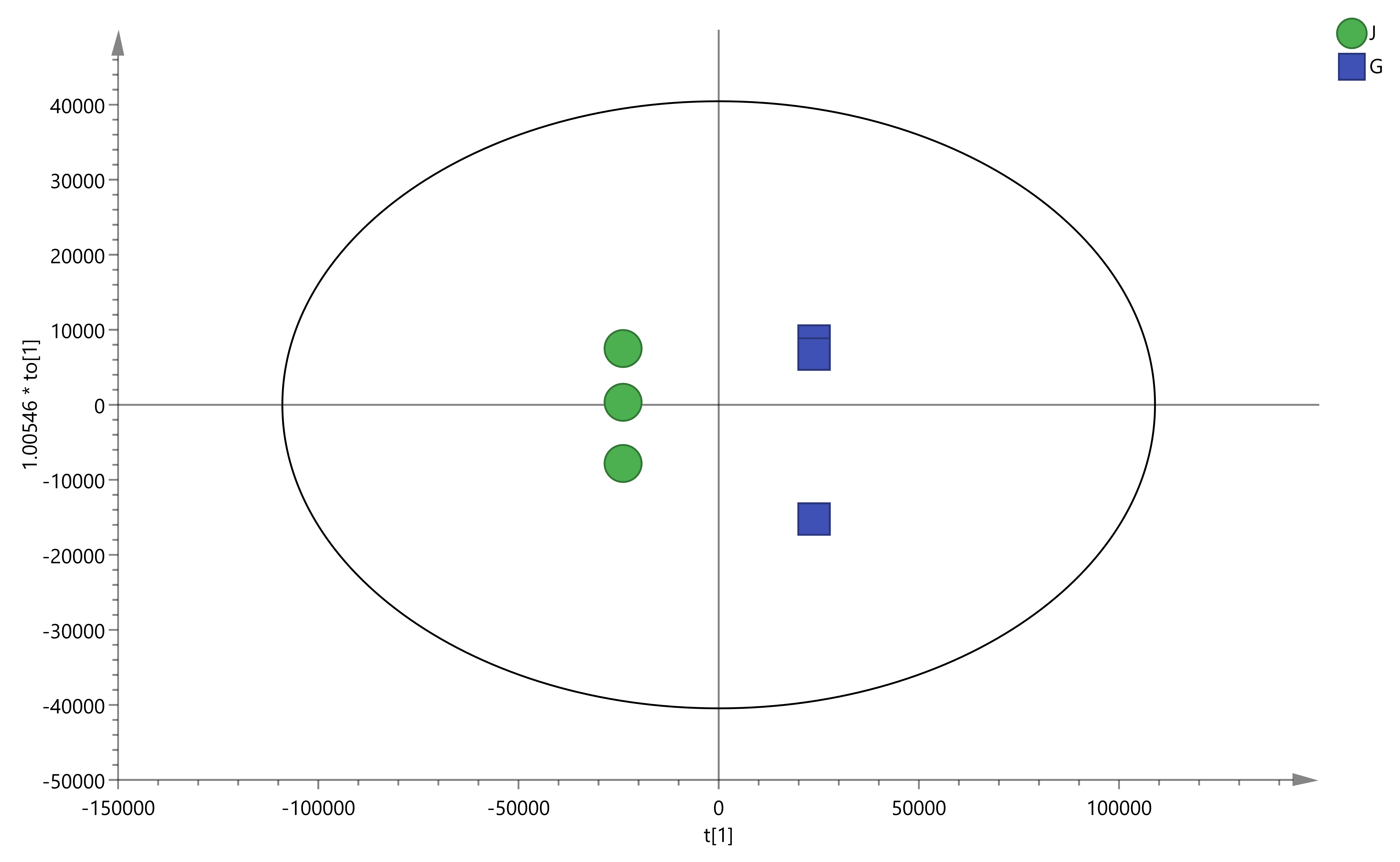

Supplement: S1 Data — (ZIP) [file pone.0353350.s002.zip › raw data/PCA/Hongju vs Huyou/OPLS-DA_J vs G.png]

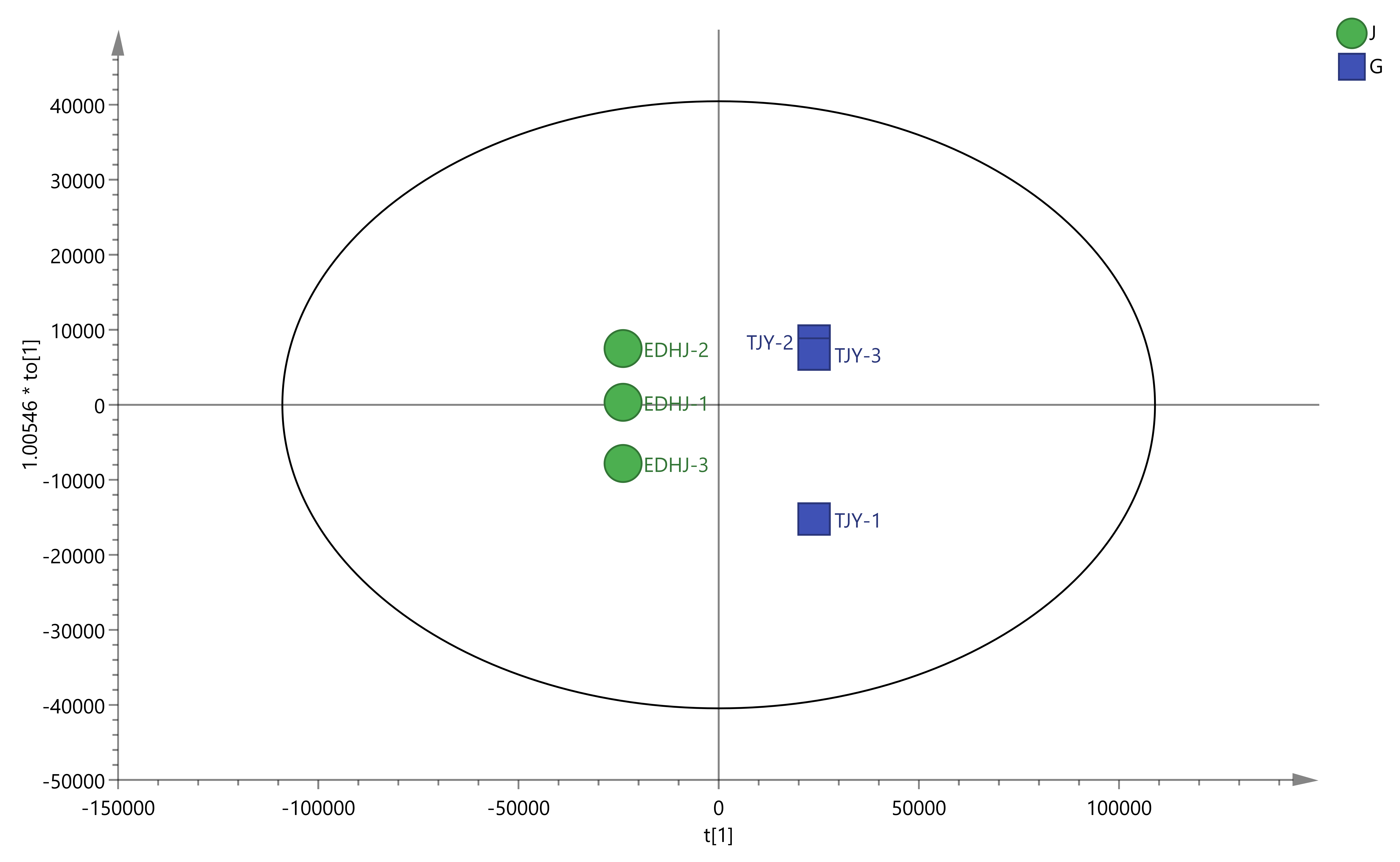

Supplement: S1 Data — (ZIP) [file pone.0353350.s002.zip › raw data/PCA/Hongju vs Huyou/OPLS-DA_J vs G_label.png]

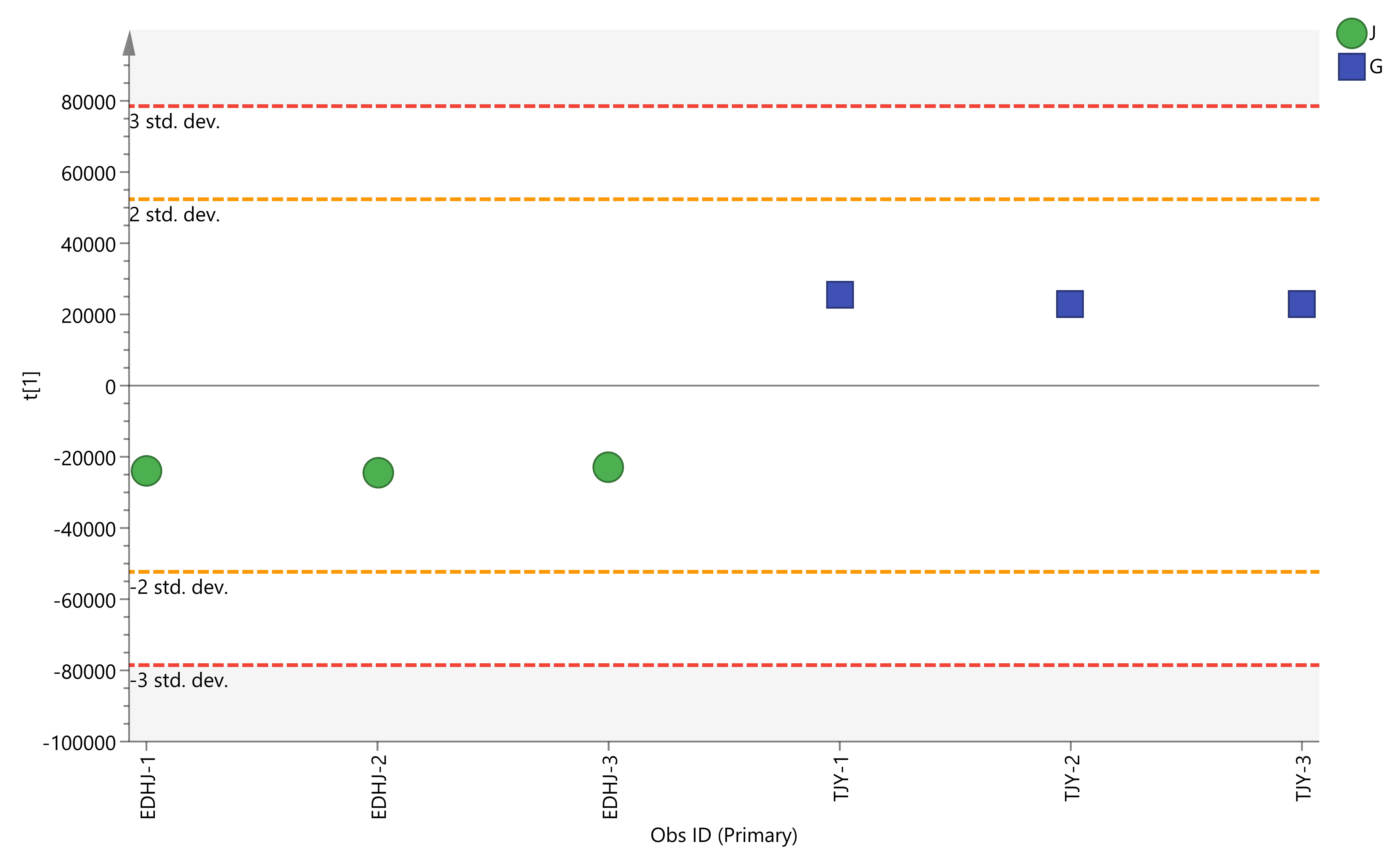

Supplement: S1 Data — (ZIP) [file pone.0353350.s002.zip › raw data/PCA/Hongju vs Huyou/PC1_Score Plot_J vs G.png]

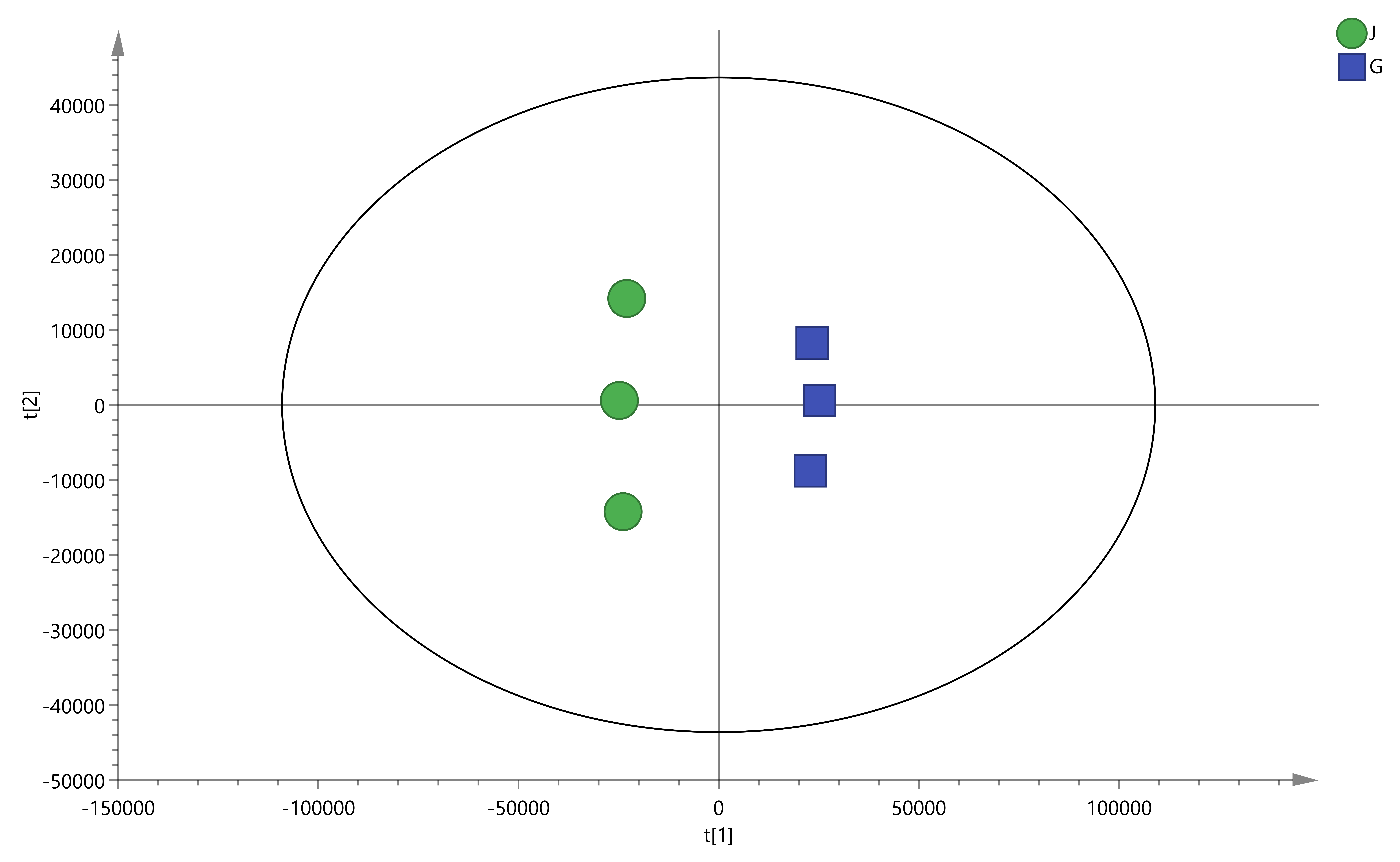

Supplement: S1 Data — (ZIP) [file pone.0353350.s002.zip › raw data/PCA/Hongju vs Huyou/PCA_J vs G.png]

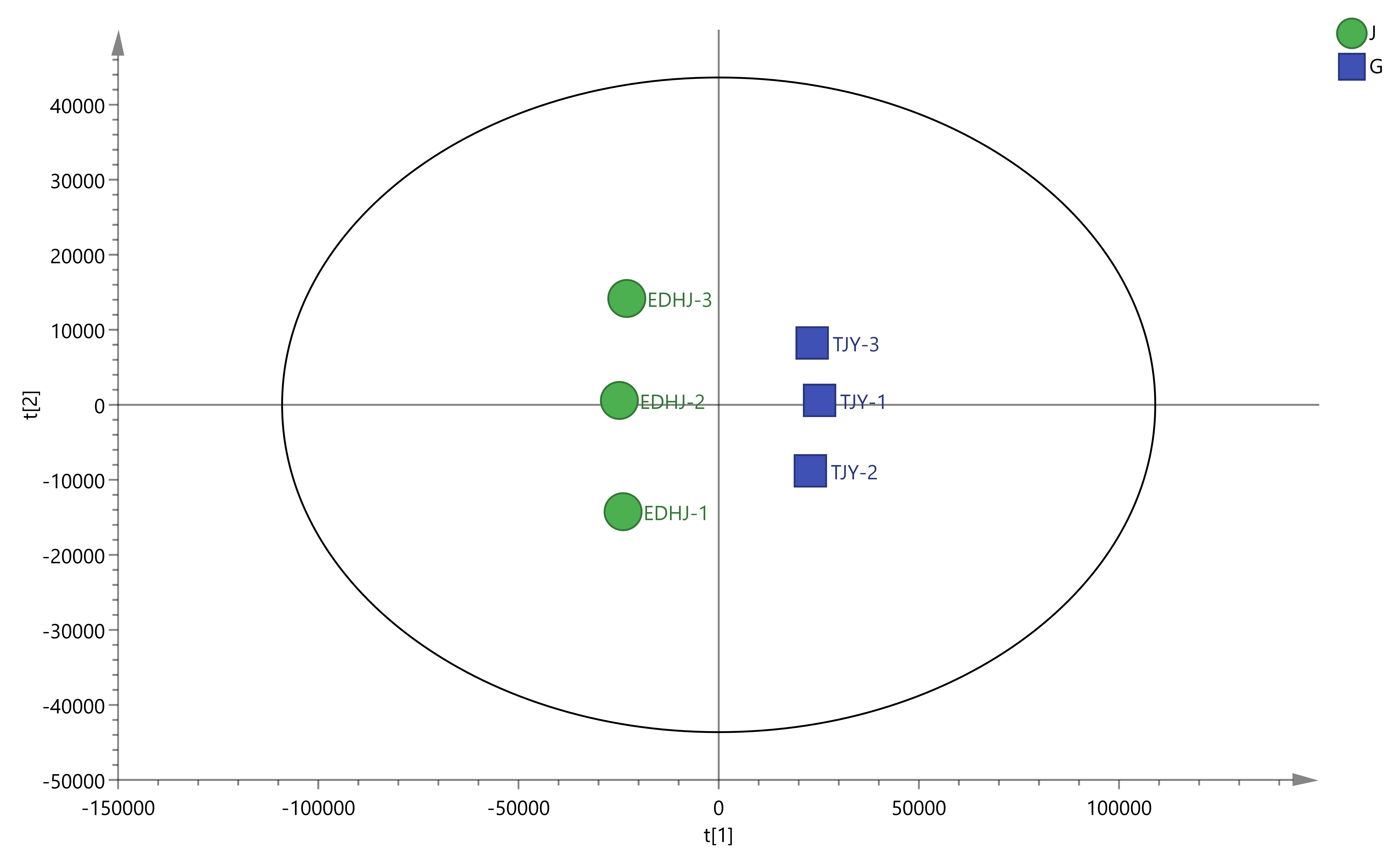

Supplement: S1 Data — (ZIP) [file pone.0353350.s002.zip › raw data/PCA/Hongju vs Huyou/PCA_J vs G_label.png]

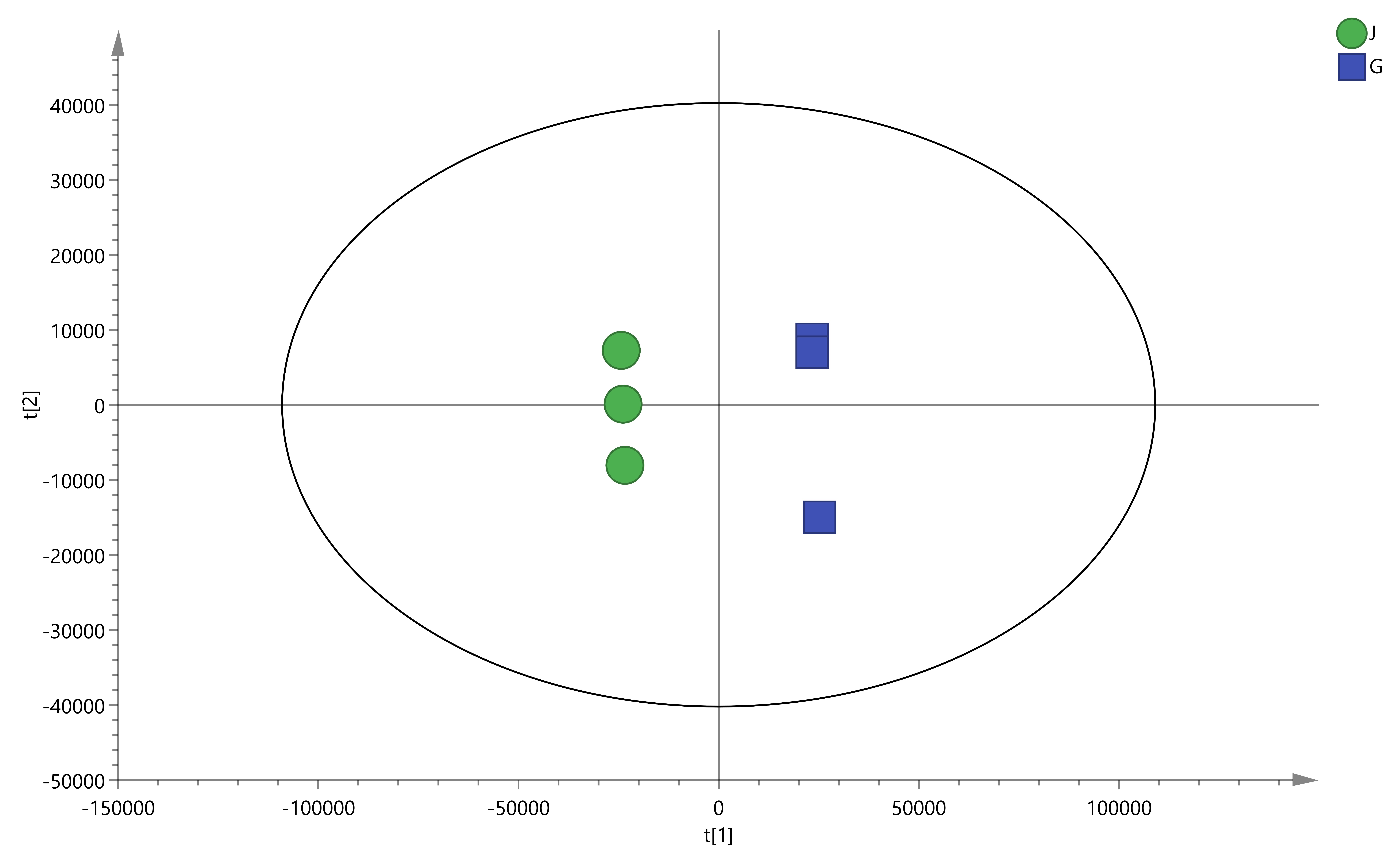

Supplement: S1 Data — (ZIP) [file pone.0353350.s002.zip › raw data/PCA/Hongju vs Huyou/PLS-DA_J vs G.png]

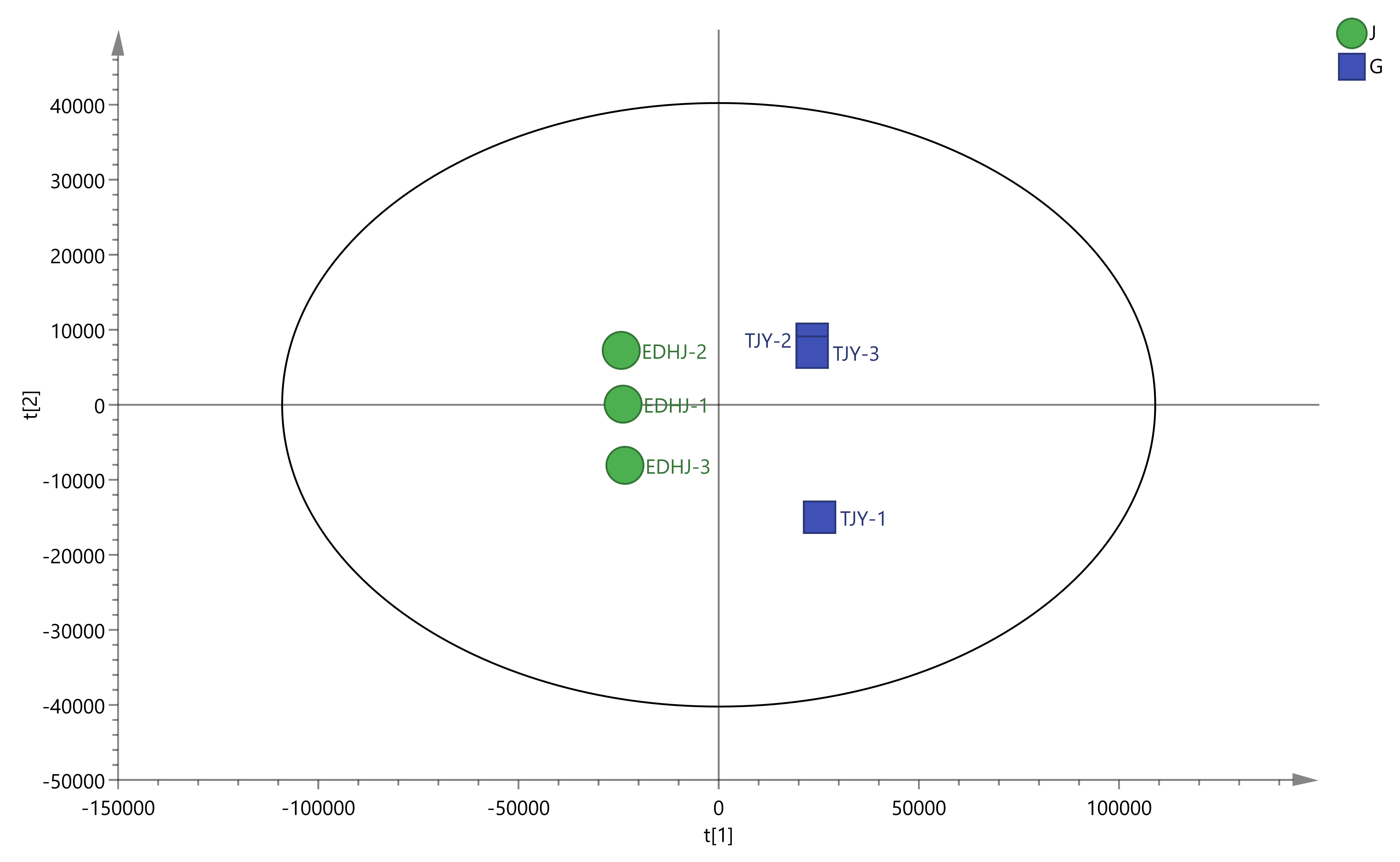

Supplement: S1 Data — (ZIP) [file pone.0353350.s002.zip › raw data/PCA/Hongju vs Huyou/PLS-DA_J vs G_label.png]

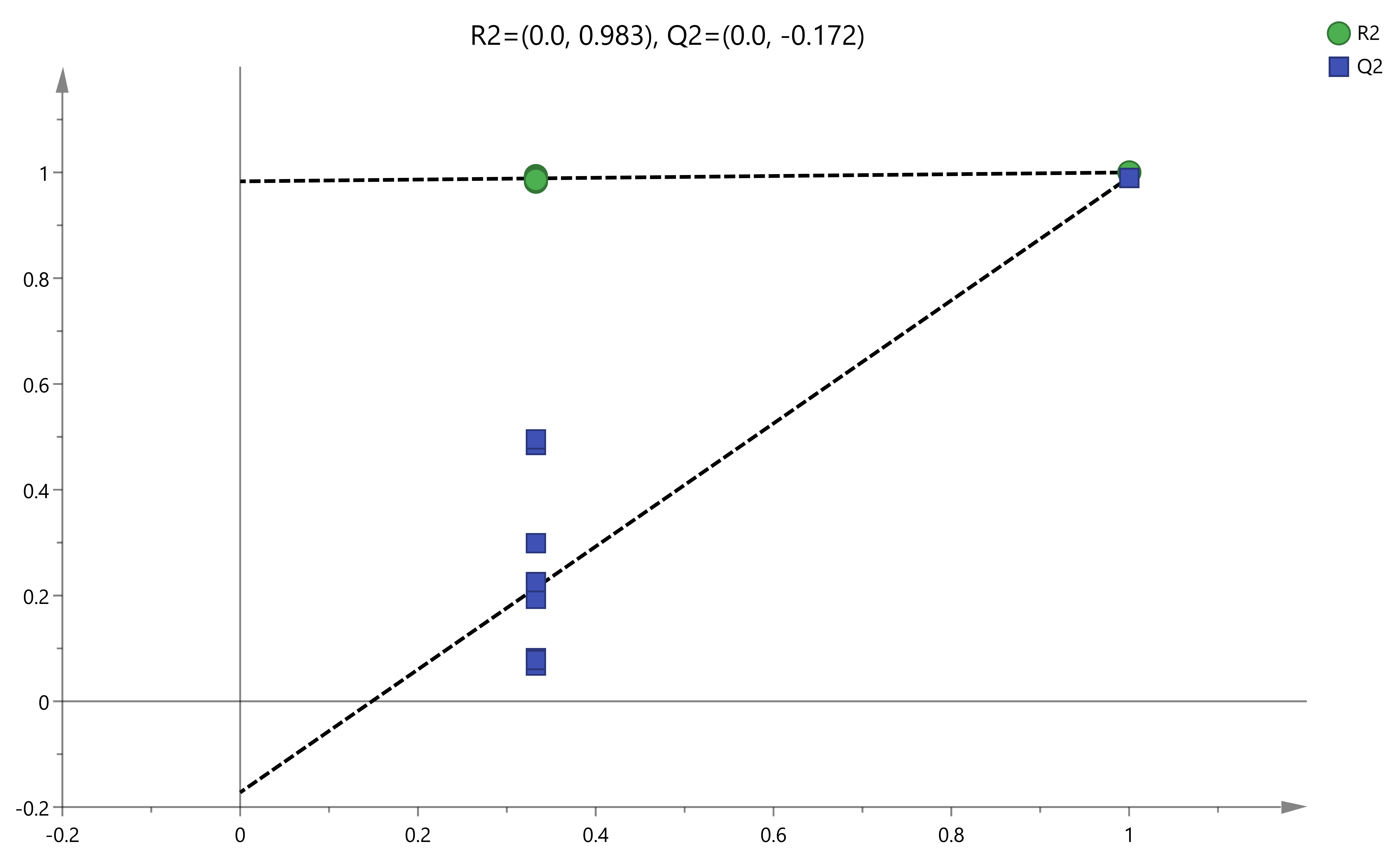

Supplement: S1 Data — (ZIP) [file pone.0353350.s002.zip › raw data/PCA/Hongju vs Huyou/Permutation-J vs G.png]

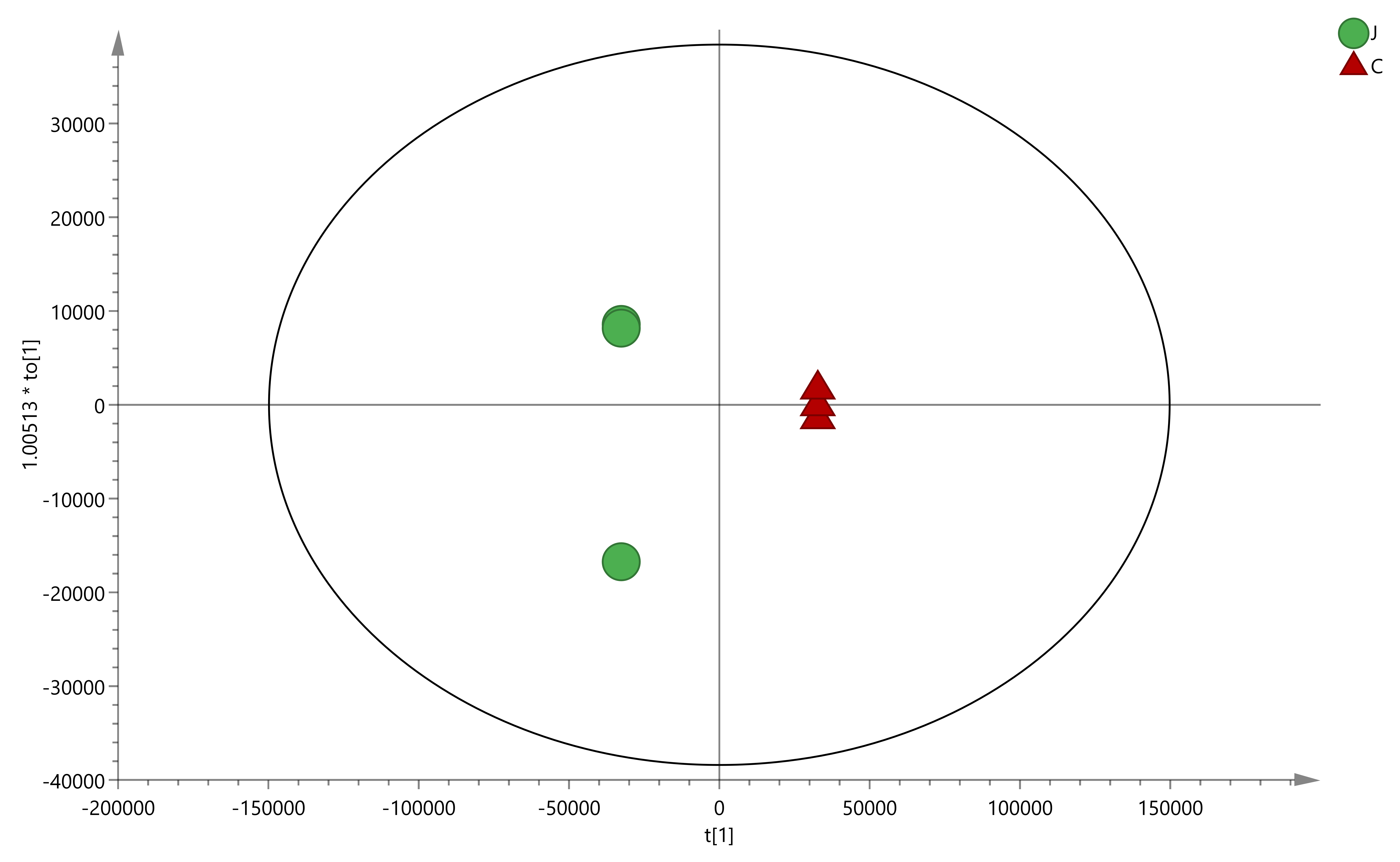

Supplement: S1 Data — (ZIP) [file pone.0353350.s002.zip › raw data/PCA/Hongju vs Xiangcheng/OPLS-DA_J vs C.png]

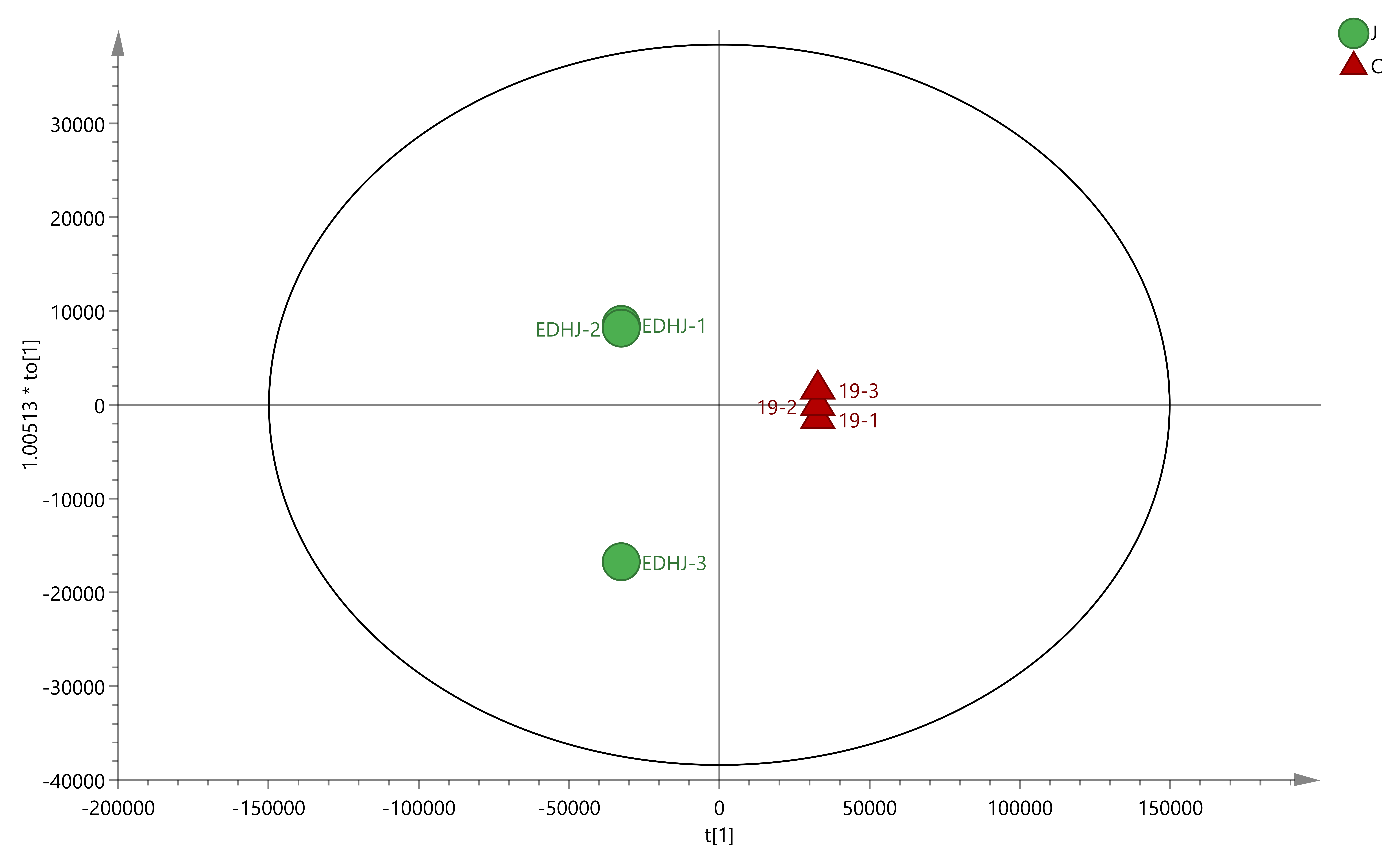

Supplement: S1 Data — (ZIP) [file pone.0353350.s002.zip › raw data/PCA/Hongju vs Xiangcheng/OPLS-DA_J vs C_label.png]

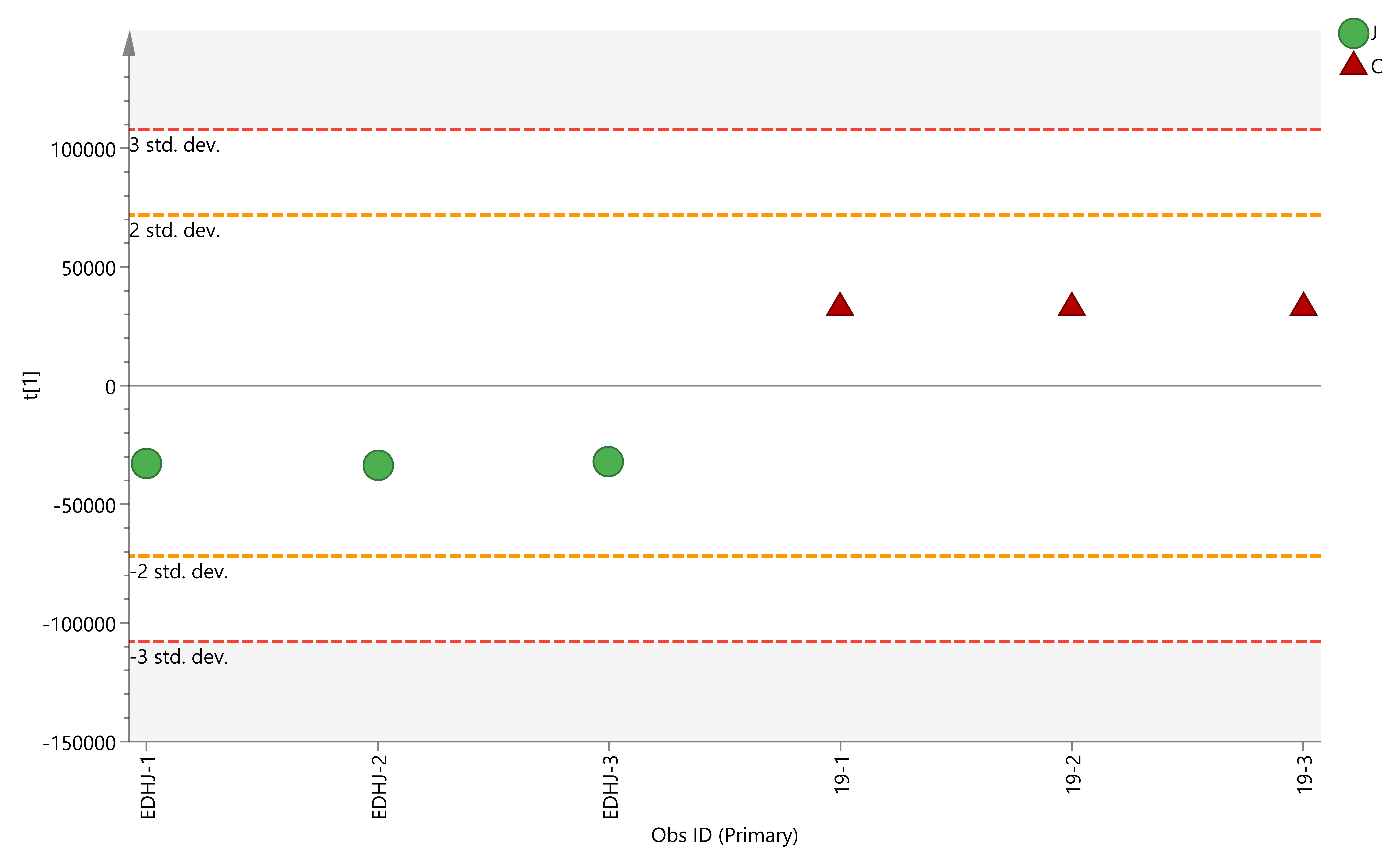

Supplement: S1 Data — (ZIP) [file pone.0353350.s002.zip › raw data/PCA/Hongju vs Xiangcheng/PC1_Score Plot_J vs C.png]

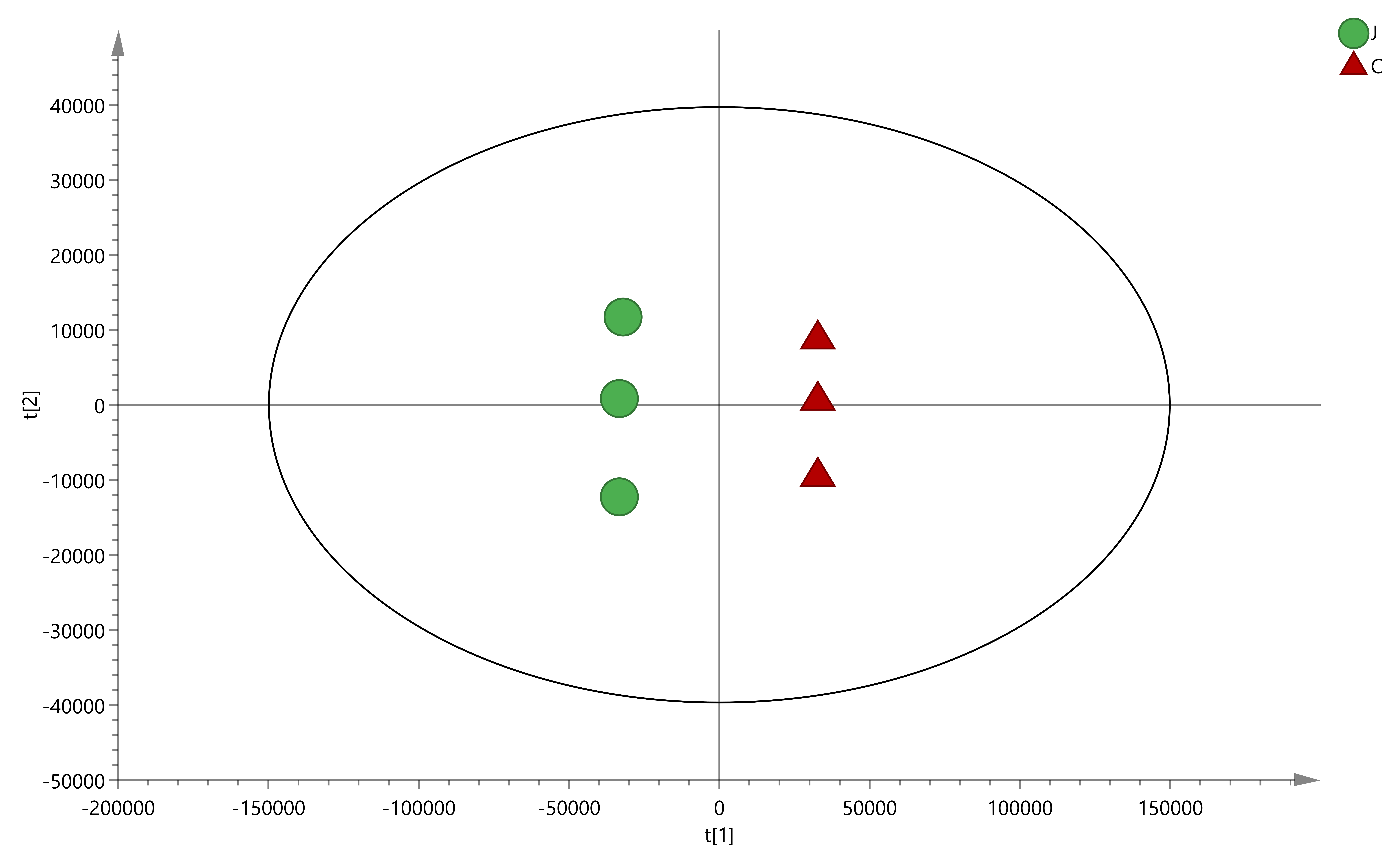

Supplement: S1 Data — (ZIP) [file pone.0353350.s002.zip › raw data/PCA/Hongju vs Xiangcheng/PCA_J vs C.png]

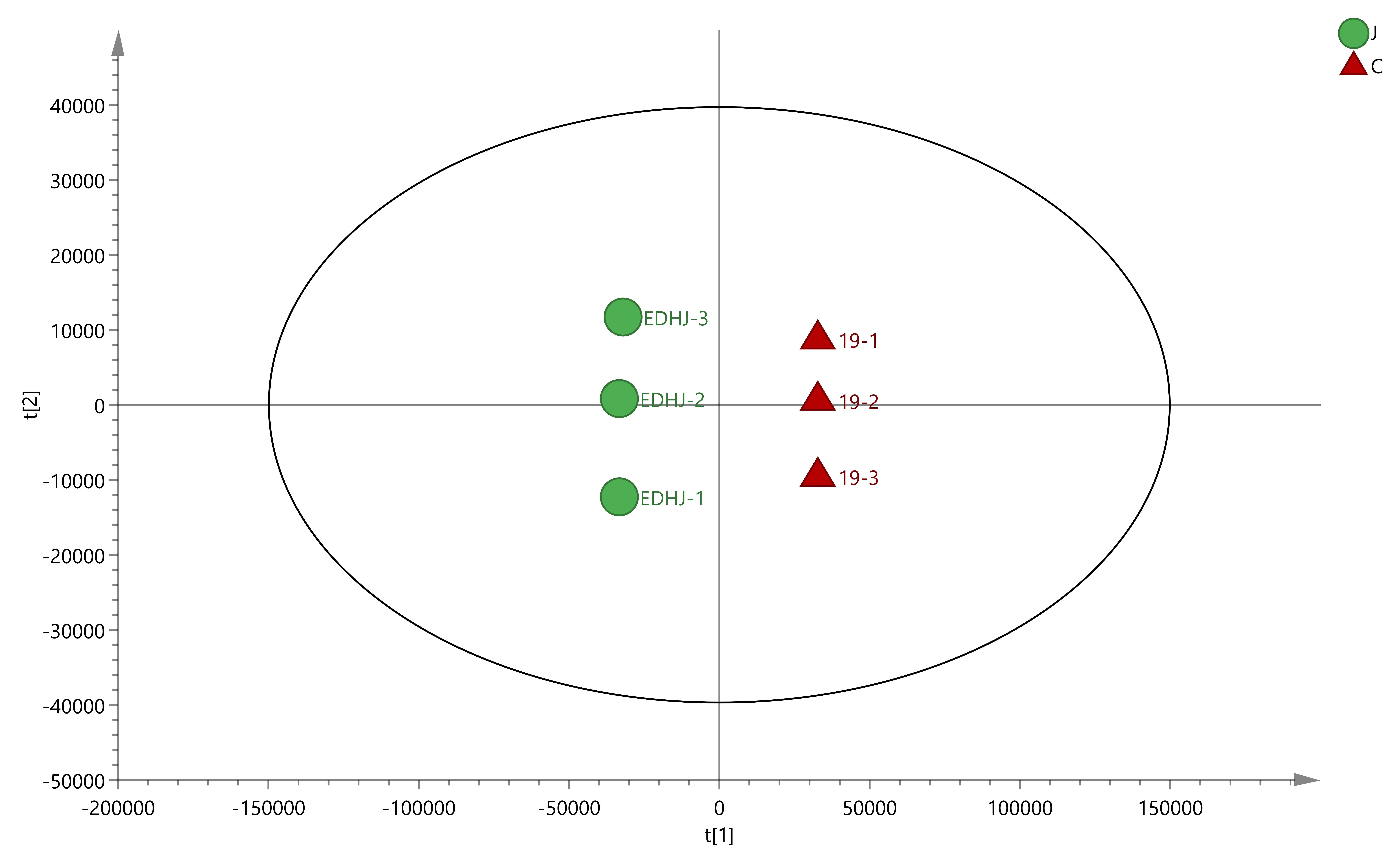

Supplement: S1 Data — (ZIP) [file pone.0353350.s002.zip › raw data/PCA/Hongju vs Xiangcheng/PCA_J vs C_label.png]

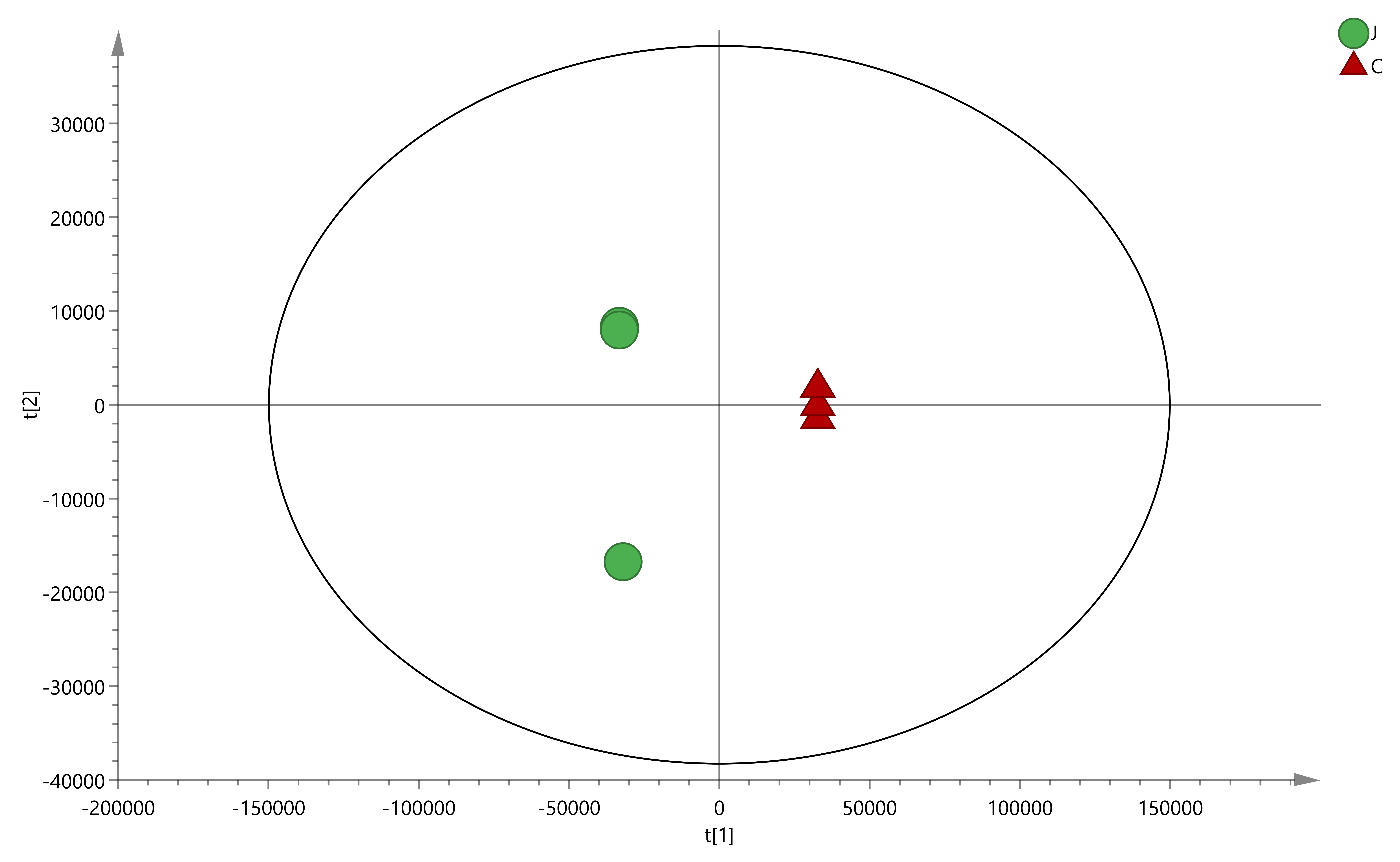

Supplement: S1 Data — (ZIP) [file pone.0353350.s002.zip › raw data/PCA/Hongju vs Xiangcheng/PLS-DA_J vs C.png]

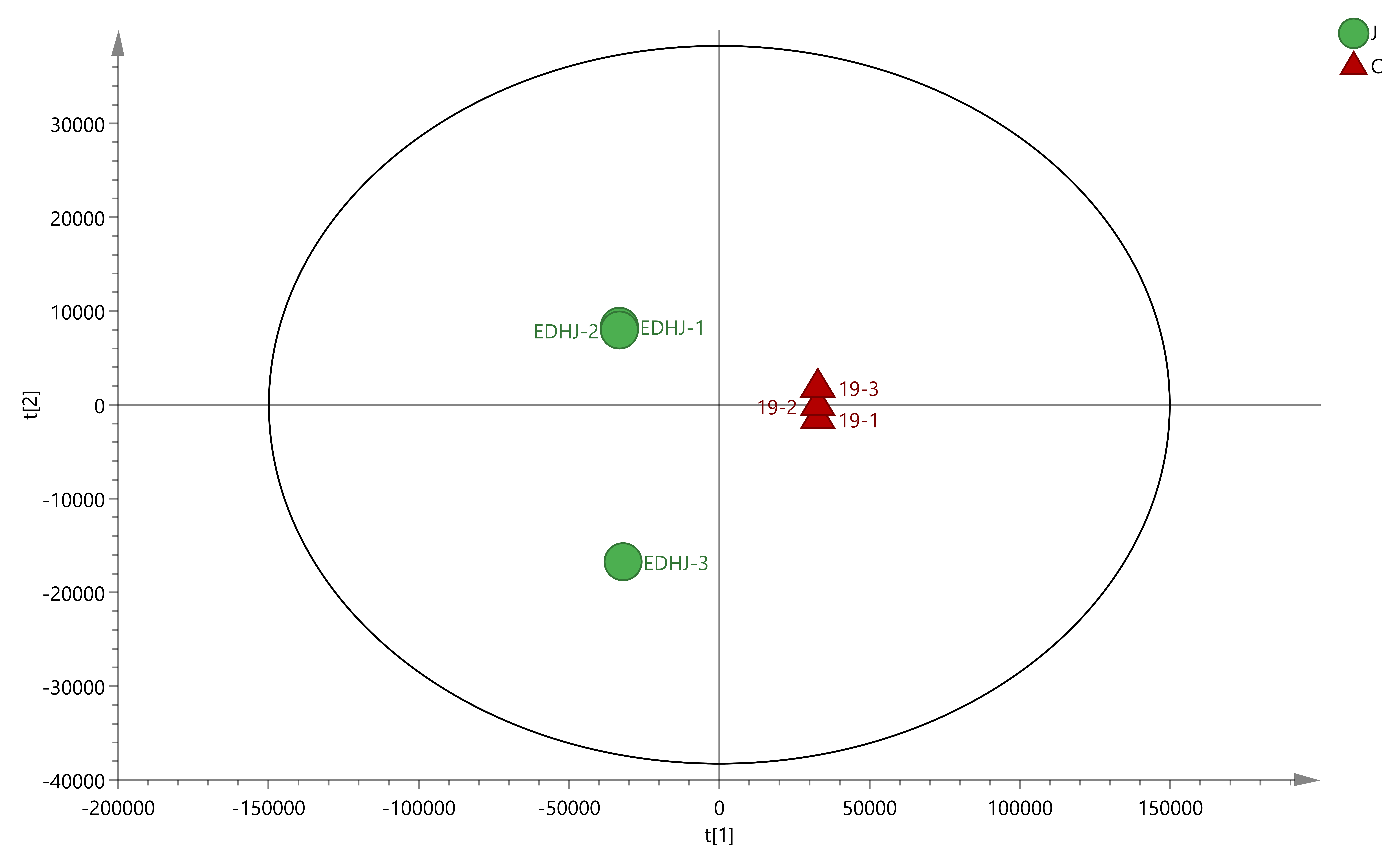

Supplement: S1 Data — (ZIP) [file pone.0353350.s002.zip › raw data/PCA/Hongju vs Xiangcheng/PLS-DA_J vs C_label.png]

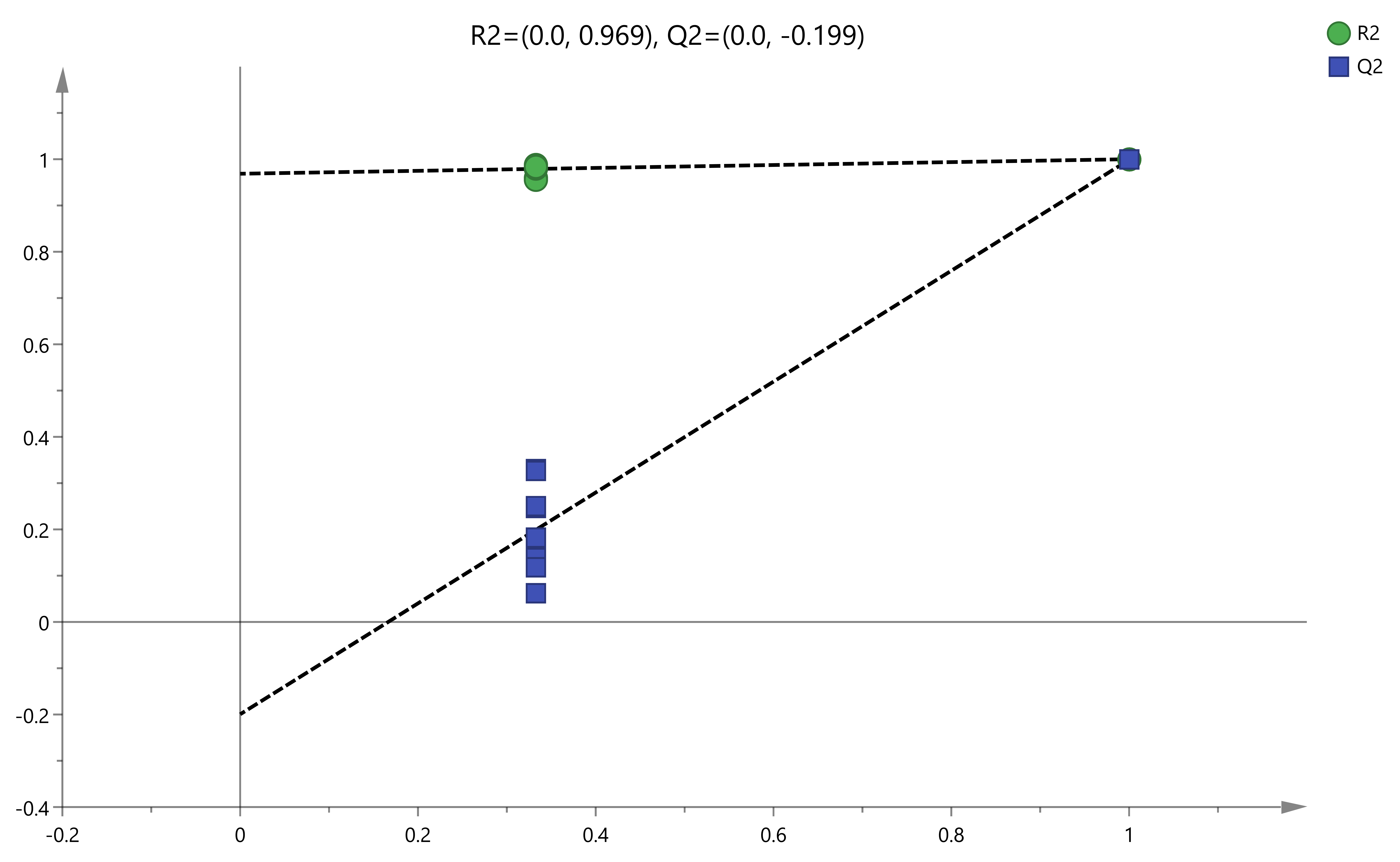

Supplement: S1 Data — (ZIP) [file pone.0353350.s002.zip › raw data/PCA/Hongju vs Xiangcheng/Permutation-J vs C.png]

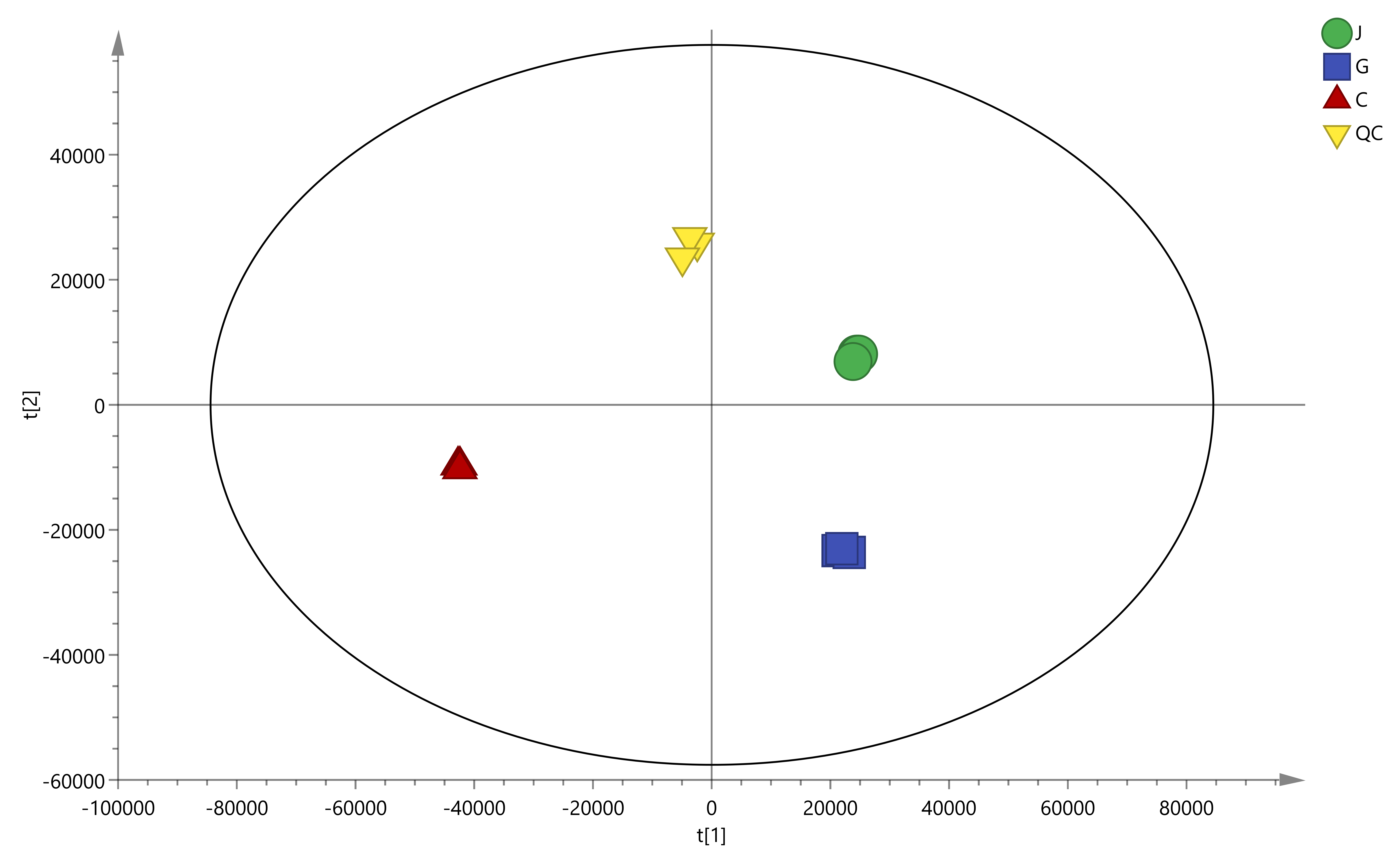

Supplement: S1 Data — (ZIP) [file pone.0353350.s002.zip › raw data/PCA/PCA_QC.png]

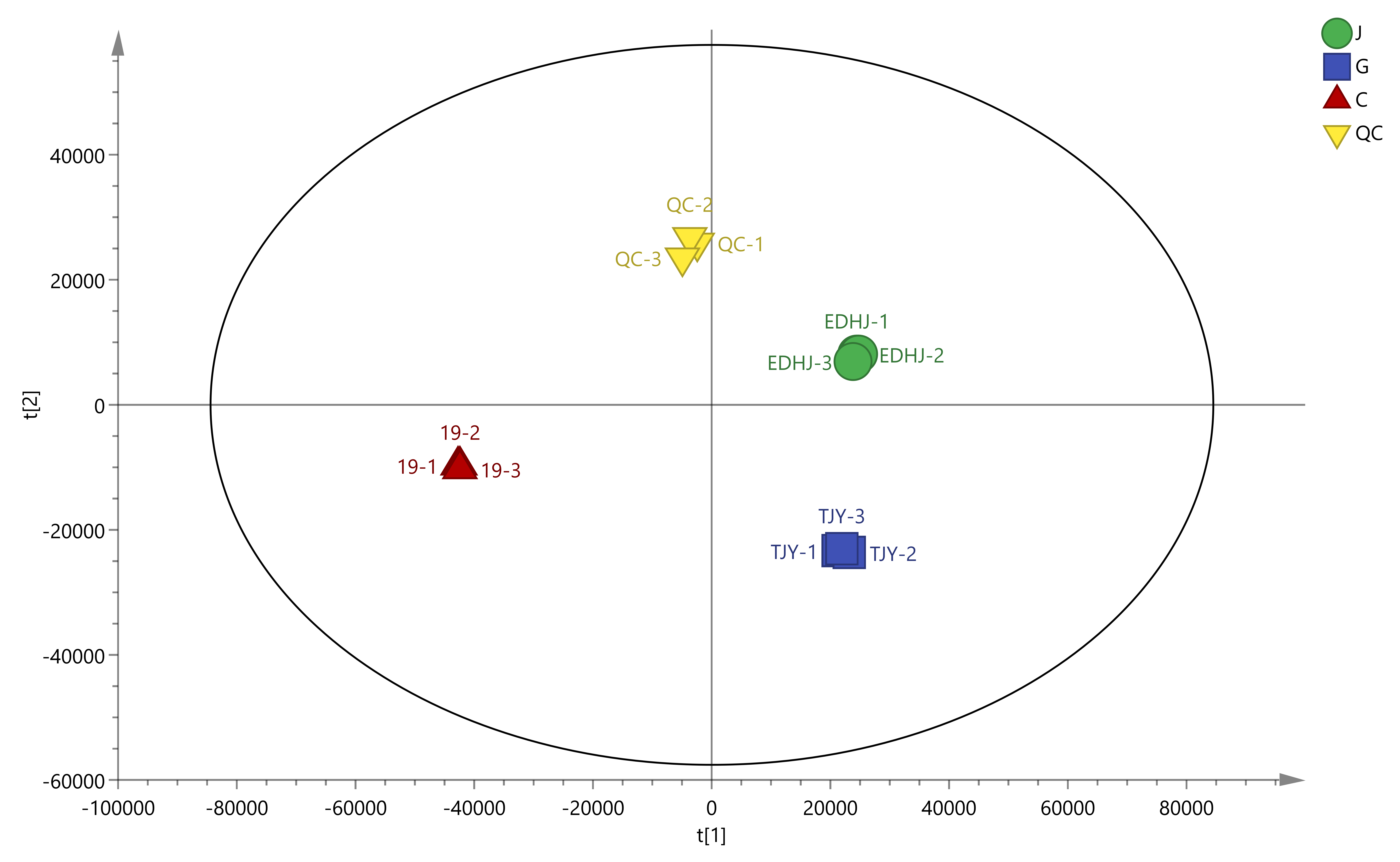

Supplement: S1 Data — (ZIP) [file pone.0353350.s002.zip › raw data/PCA/PCA_QC_label.png]

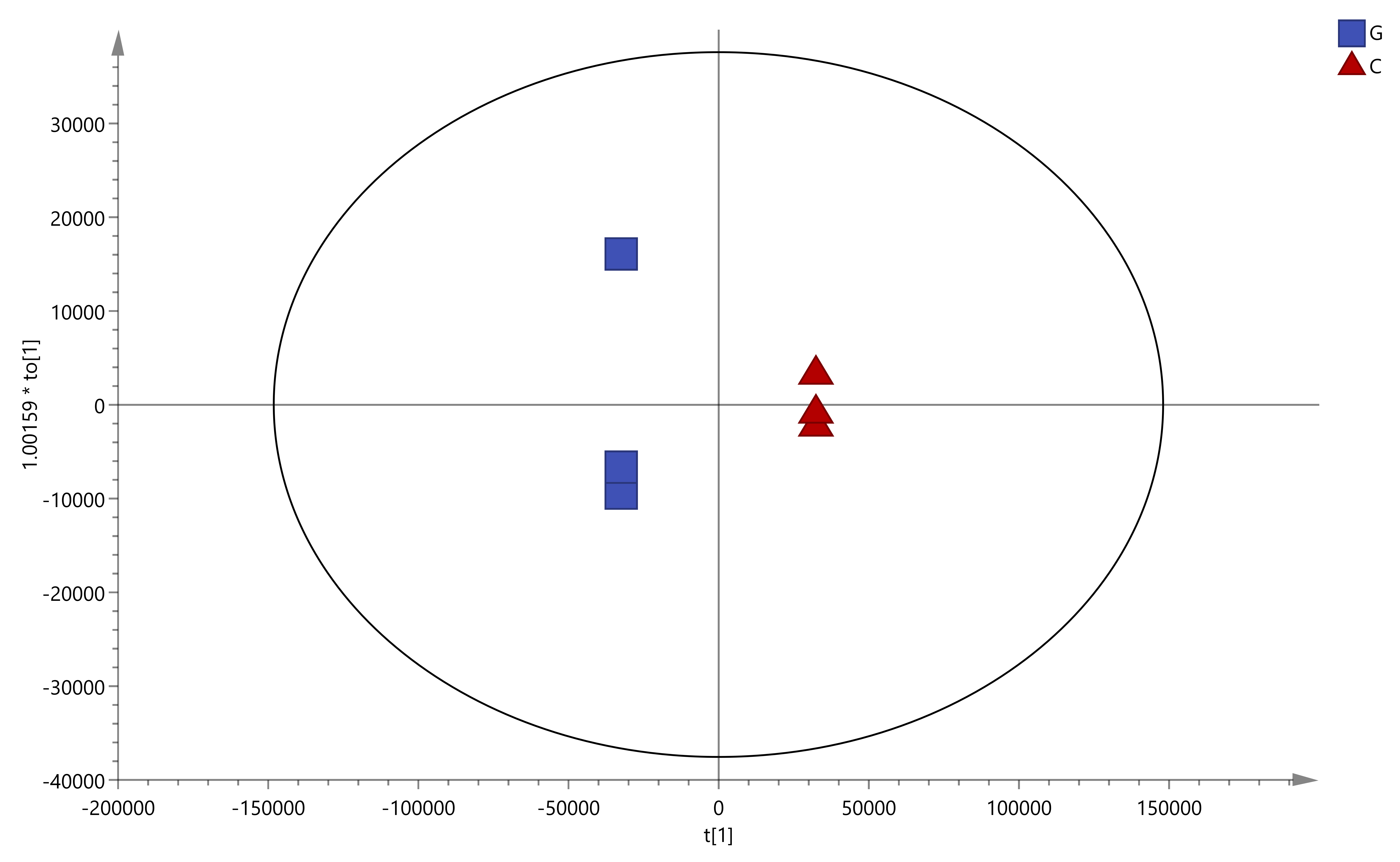

Supplement: S1 Data — (ZIP) [file pone.0353350.s002.zip › raw data/PCA/Xiangcheng vs Huyou/OPLS-DA_C vs G.png]

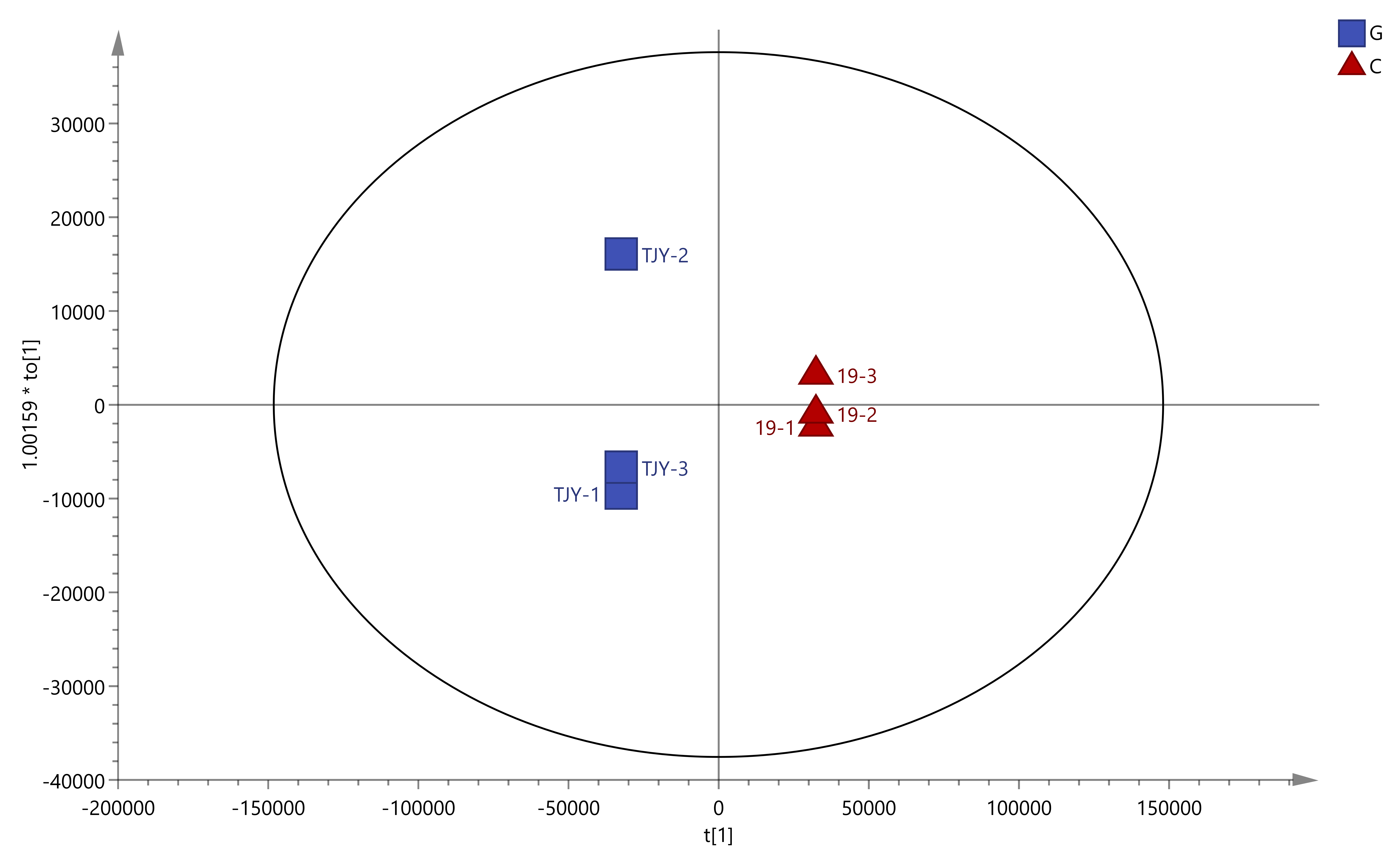

Supplement: S1 Data — (ZIP) [file pone.0353350.s002.zip › raw data/PCA/Xiangcheng vs Huyou/OPLS-DA_C vs G_label.png]

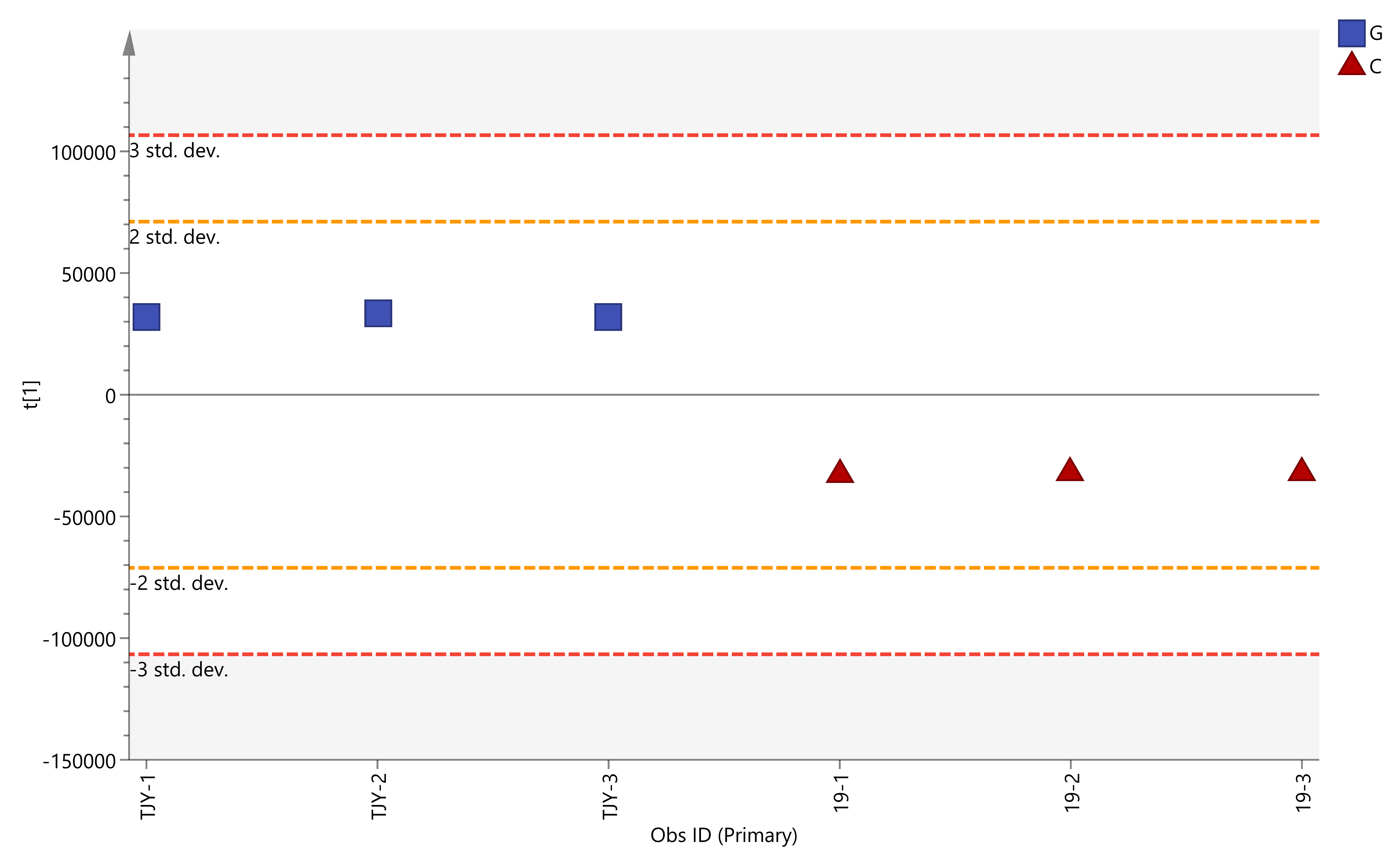

Supplement: S1 Data — (ZIP) [file pone.0353350.s002.zip › raw data/PCA/Xiangcheng vs Huyou/PC1_Score Plot_C vs G.png]

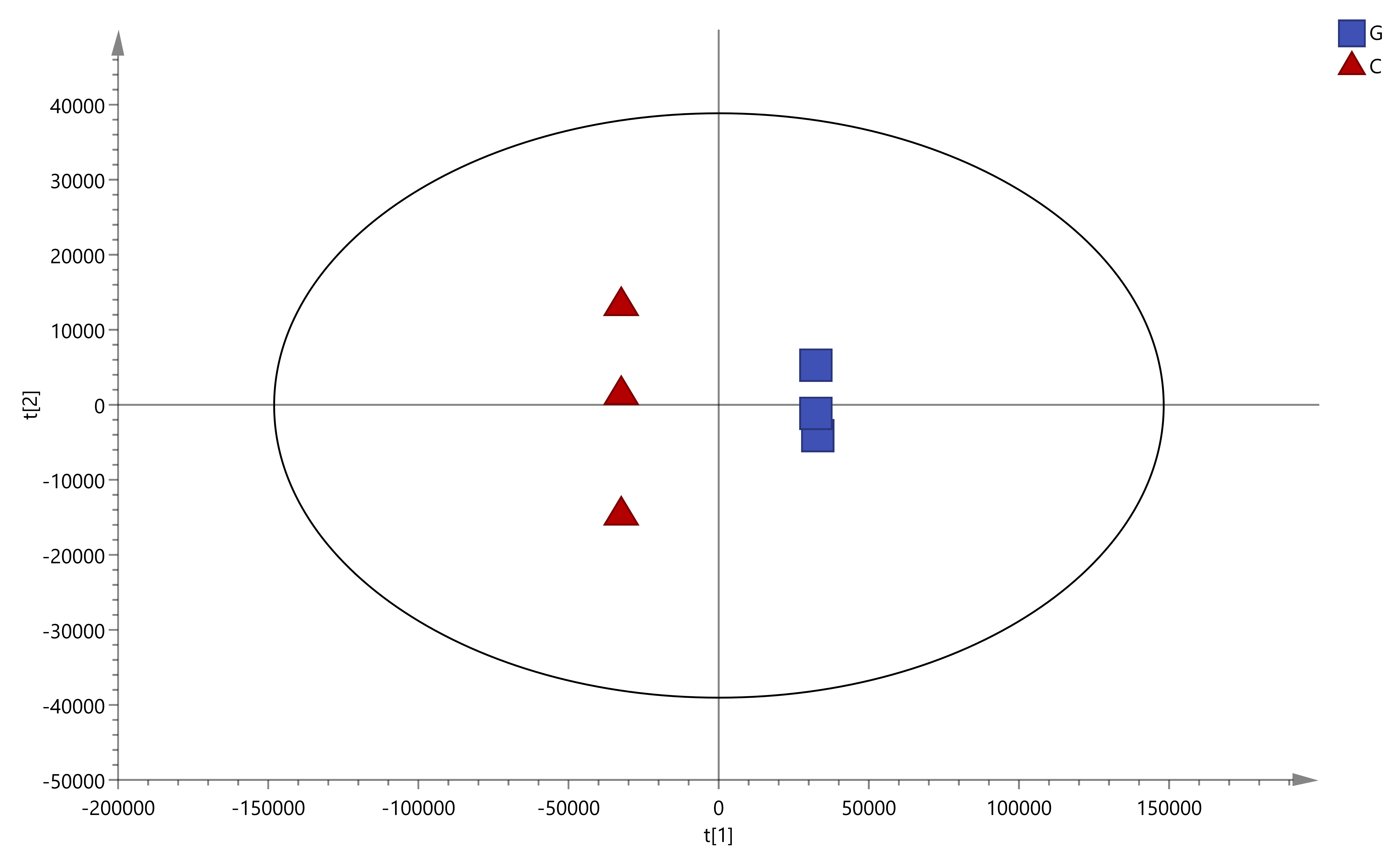

Supplement: S1 Data — (ZIP) [file pone.0353350.s002.zip › raw data/PCA/Xiangcheng vs Huyou/PCA_C vs G.png]

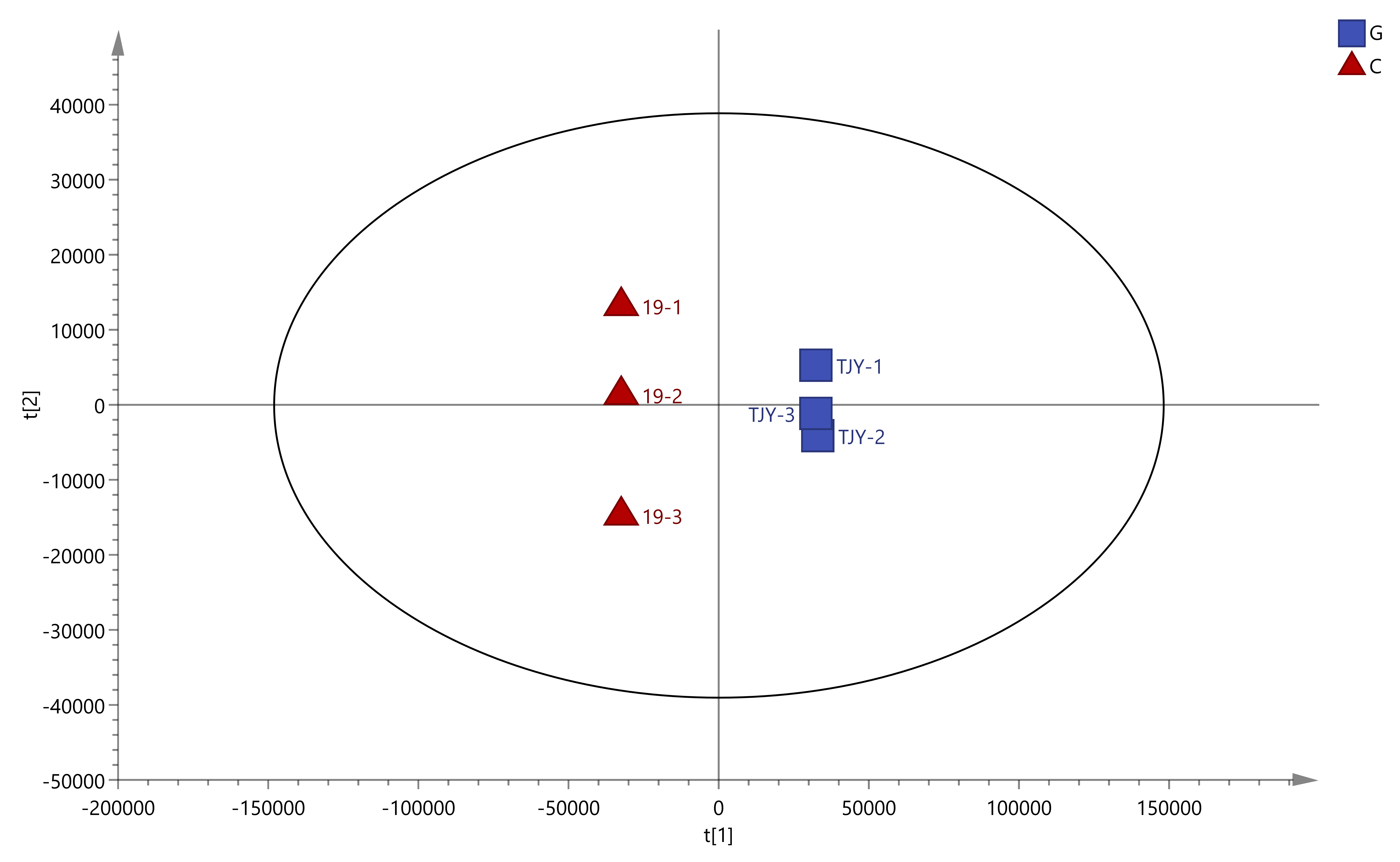

Supplement: S1 Data — (ZIP) [file pone.0353350.s002.zip › raw data/PCA/Xiangcheng vs Huyou/PCA_C vs G_label.png]

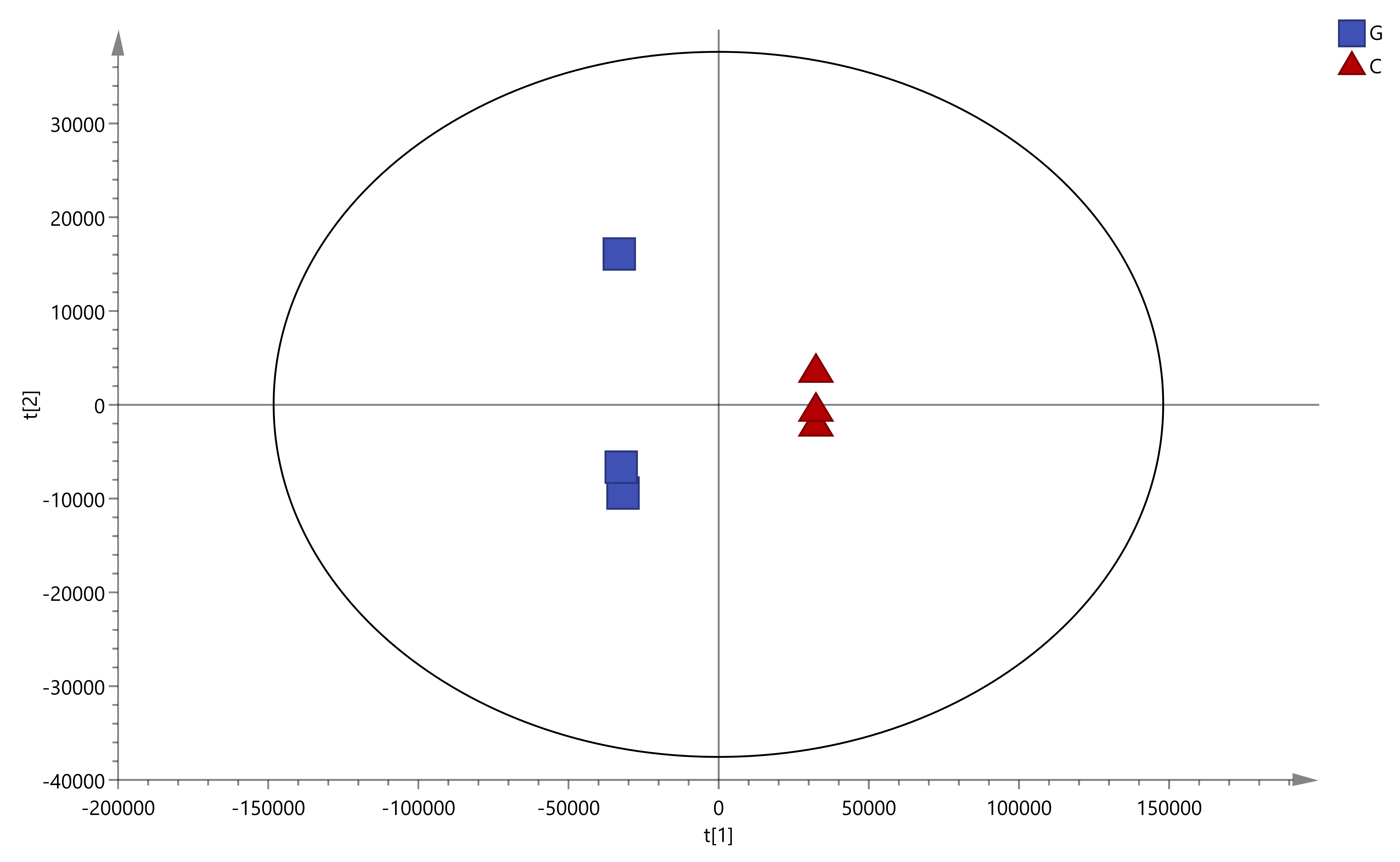

Supplement: S1 Data — (ZIP) [file pone.0353350.s002.zip › raw data/PCA/Xiangcheng vs Huyou/PLS-DA_C vs G.png]

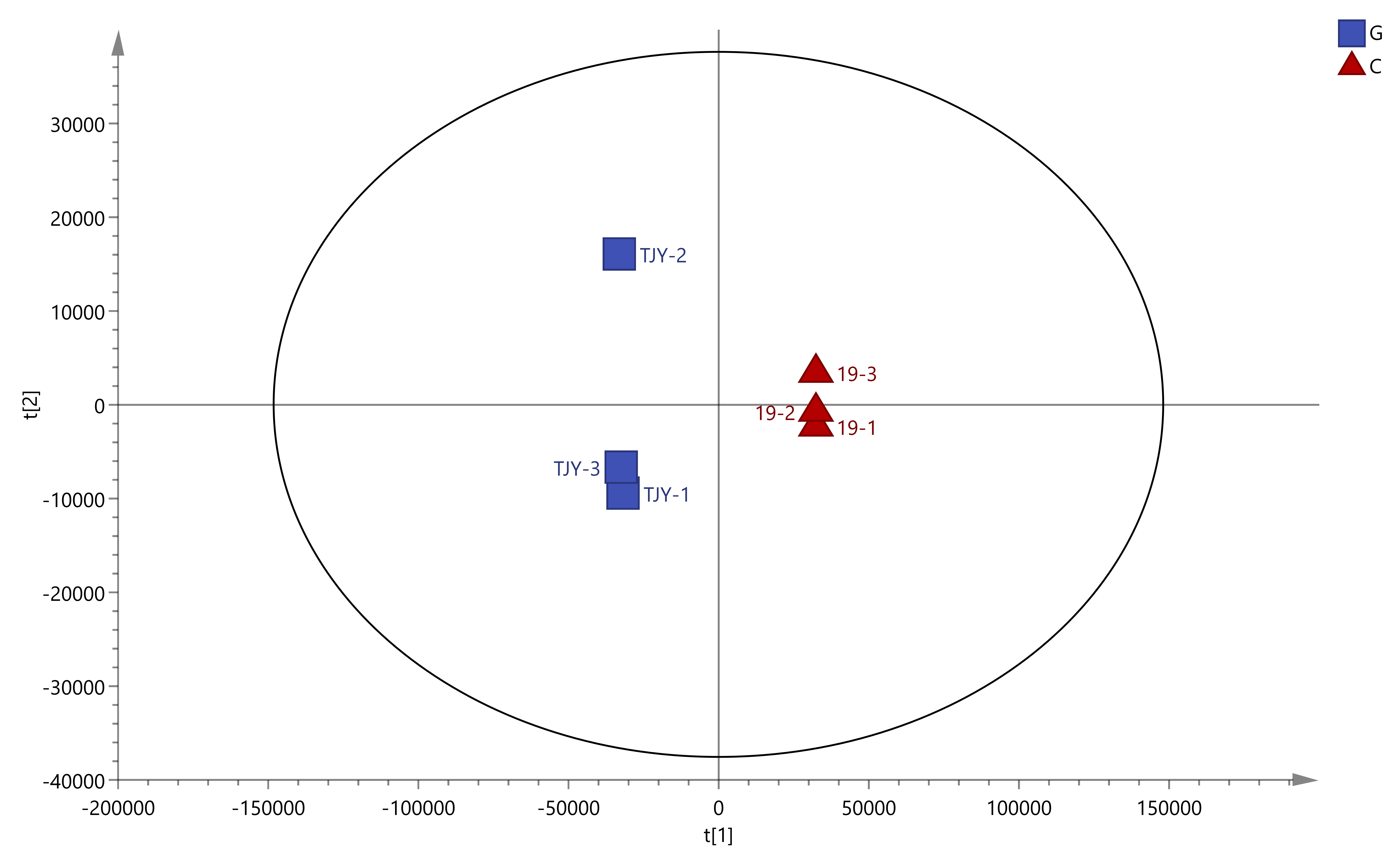

Supplement: S1 Data — (ZIP) [file pone.0353350.s002.zip › raw data/PCA/Xiangcheng vs Huyou/PLS-DA_C vs G_label.png]

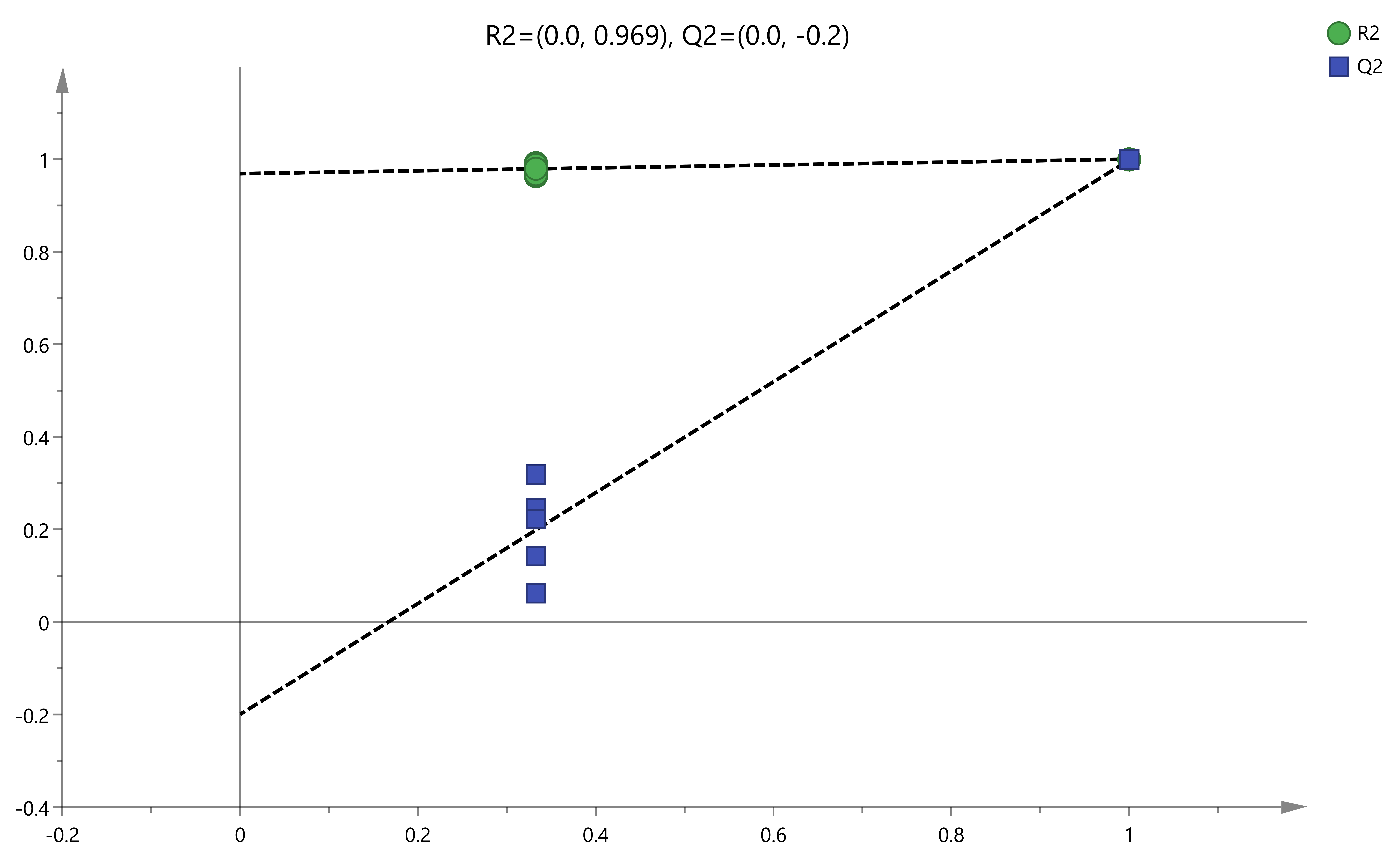

Supplement: S1 Data — (ZIP) [file pone.0353350.s002.zip › raw data/PCA/Xiangcheng vs Huyou/Permutation-C vs G.png]
